# Electrospun Polymer Nanofibers: Processing, Properties, and Applications

**DOI:** 10.3390/polym15010065

**Published:** 2022-12-23

**Authors:** Abdulhamid Al-Abduljabbar, Irfan Farooq

**Affiliations:** Department of Mechanical Engineering, King Saud University, Riyadh 11421, Saudi Arabia

**Keywords:** polymer nanofibers, electrospinning, polymer processing, mechanical properties, biomedical application, energy storage separation, composite materials functional nanofiber

## Abstract

Electrospun polymer nanofibers (EPNF) constitute one of the most important nanomaterials with diverse applications. An overall review of EPNF is presented here, starting with an introduction to the most attractive features of these materials, which include the high aspect ratio and area to volume ratio as well as excellent processability through various production techniques. A review of these techniques is featured with a focus on electrospinning, which is the most widely used, with a detailed description and different types of the process. Polymers used in electrospinning are also reviewed with the solvent effect highlighted, followed by a discussion of the parameters of the electrospinning process. The mechanical properties of EPNF are discussed in detail with a focus on tests and techniques used for determining them, followed by a section for other properties including electrical, chemical, and optical properties. The final section is dedicated to the most important applications for EPNF, which constitute the driver for the relentless pursuit of their continuous development and improvement. These applications include biomedical application such as tissue engineering, wound healing and dressing, and drug delivery systems. In addition, sensors and biosensors applications, air filtration, defense applications, and energy devices are reviewed. A brief conclusion is presented at the end with the most important findings and directions for future research.

## 1. Introduction

Interest in polymer nanofibers has increased rapidly in the recent few decades. The main drivers include not only the improved properties due to high aspect ratio and surface area to volume, but also the manufacturability and diversity of applications. Some sources [1] attribute nanofibers’ class to fibers with a ratio of length to thickness in the order of one thousand. Others classify nanofibers as nanomaterials that have at least one dimension of 100 nm or less [2,3]. Since the cross-section area of the nanofiber is meant here, the nanoscale is actually in two dimensions. A nanofiber with a diameter of ∼100 nm can have a specific surface area up to 1000 m^2^/g [4].

Although meso- and nano-porous materials, such as adsorbent granules and powders, can achieve large surface areas exceeding 2000 m^2^/g, fibers are easier to handle and more suitable for use in numerous crucial applications in contrast with powders. The benefit of large surface areas in organic fibers is frequently coupled with the flexibility of surface functionality, which can be utilized for a variety of applications, for example, biomedical applications, effective filtration, smart textiles, and improved fiber–matrix interaction for composite reinforcement applications [5]. Polymeric materials are widely utilized to manufacture nanofibers because of many attractive features and properties such as low cost, light weight, easy and diverse processing techniques, and flexibility of utilization and recyclability [6,7,8]. Combined characteristics found in polymer nanofibers make them strong candidates for such diverse and important applications [9,10]. Polymeric fibers’ remarkable features improve as the fiber diameter decreases from micrometer to submicron or nanometer. These include an extremely high surface area to volume ratio, pliability in surface functionalities, and superior mechanical properties compared to other materials. Due to their exceptional characteristics, polymer nanofibers are favored for a variety of applications [11].

Nanofibers can be produced by selecting the proper combination of polymers and additives and by using appropriate production techniques based on several essential characteristics that have an impact on meeting the criteria of the intended particular application area [12]. Among the different techniques which have been developed to process polymer nanofibers are phase separation or inversion [13,14], spinneret-based tunable engineered parameters [15], self-assembly polymerization [16], template synthesis [17], hot stretching [18], and electrospinning [19,20]. A comparison of different techniques for production of polymer nanofibers including electrospinning methods is shown in Table 1. Electrospinning is considered as the most prominent method to process polymer nanofibers [21]. Electrospinning is a relatively simple process that uses diverse polymers. Moreover, it produces long continuous nanofibers, and it can feasibly generate aligned nanofibers. Using this technique, a huge range of polymers can be processed to successfully reduce fiber diameter to nanometer scale, with the possibility of scaling up production [10,11,22].

**Figure 1:**
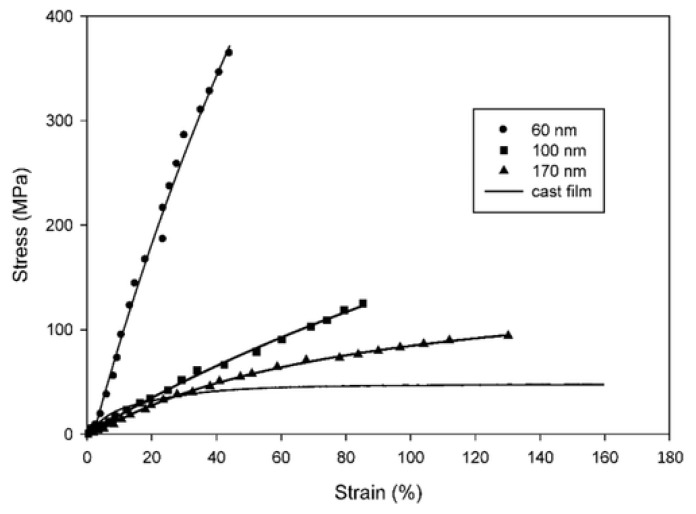
A typical plot of stress–strain relationship for nylon-6 nanofibers of different diameters, with the reference being the cast film. Reproduced from [26] with the permission of Elsevier.

Attractive properties of electrospun nanofibers include the extremely high specific surface area, high porosity (typically 90%), light weight, controllable pore size, flexibility in surface functionalities, large permeability, excellent mechanical properties, high aspect ratio, and length up to many centimeters [11,23,24]. Electrospinning offers a top-down approach, in contrast to many bottom-up techniques used to create nanostructures, which results in cost effectiveness and simple processability [25]. Due to less material defects and greater molecular orientation, electrospun nanofibers generally appear to have somewhat high mechanical characteristics, as compared to their bulk material (Figure 1) [26].

In the spinning technique, electrospun nanofibers experience significant elongation, with a draw ratio up to $10^{4}$ and a strain rate of $10^{5}$ s^−1^ [27,28]. Due to high strain and strong shear forces, highly aligned molecular chains develop along the fiber axis. However, in the spinning process, polymer chains are relaxed with sufficient molecular adjustment in fibers [23,25,29]. Additionally, it has been discovered that electrospun nanofibers are birefringent, which indicates molecular alignment [24].

In this paper, we review the electrospinning process, types of polymers used in electrospinning nanofibers, the processing parameters related to electrospinning equipment and environmental effects on electrospun polymer nanofibers. Then, we cover their mechanical, electrical, chemical, and optical properties and review various techniques to measure them. We also cover mathematical modeling of nanofibers’ behavior. Finally, we conclude by discussing the most prominent applications in different fields.

## 2. The Electrospinning Process

### 2.1. A Brief History

**Figure 2:**
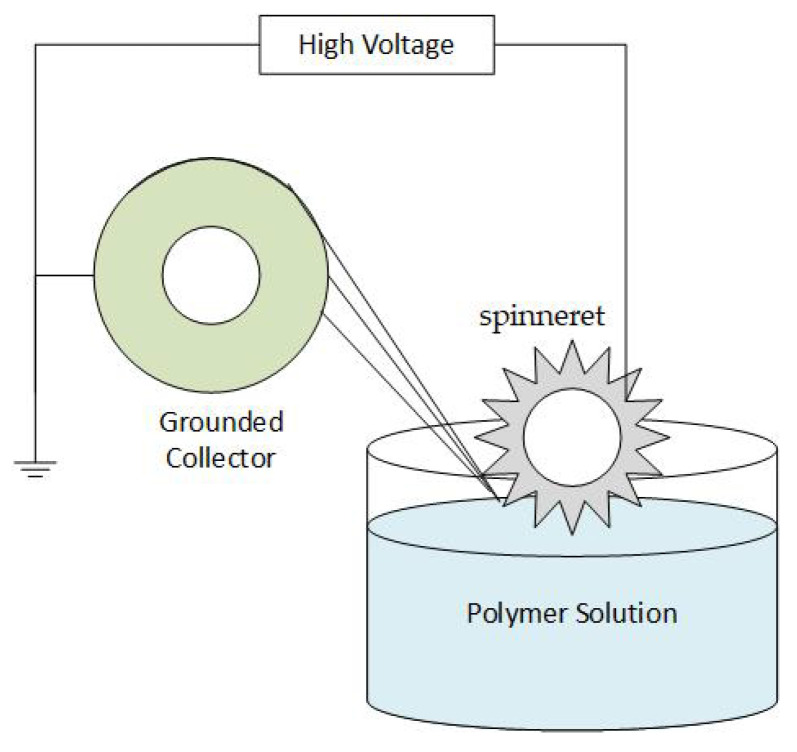
Schematic of Formhals’ electrospinning apparatus.

Electrospinning is a facile technique to fabricate extremely thin fibers from a wide range of materials, including polymers, ceramics, composites, etc. More than two hundred distinct types of polymers have been electrospun [4,10,30,31]. Although the name “electrospinning” (derived from “electrostatic spinning”) has only been in use since 1994, its basic concept has existed for more than sixty years. In 1934, Anton Formhals presented the pivotal patent in which he elaborated on the electrospinning of plastics. In a series of patents submitted between 1934 and 1944, Formhals explained the experimental setup for creating polymer filaments by utilizing electrostatic force [32]. To prepare polymer filaments, a cellulose acetate solution was introduced to an electric field. One electrode was dipped into the solution, and the second was placed on a collector. The charged solution jets were propelled from a metal spinneret having a tiny hole and evaporated to produce fibers, which were gathered at the electrically grounded collector. Figure 2 shows a diagram of the basic setup. Later, in 1971, Baumgarten developed a device to electrospin acrylic fibers with diameters ranging from 0.05 to 1.1 microns [33].

In this procedure, a stainless-steel capillary tube was utilized to suspend a droplet of polymer solution, and feed rate was controlled by infusion pump while maintaining a constant droplet size. The capillary tube was connected to a high-voltage DC current, and fibers were collected at a specific distance on a grounded metal screen. However, this innovation, which was followed by an additional 12 patents, went mostly unrecognized. The electrospinning procedure, essentially like that described by Baumgarten, has garnered significantly more attention since the 1990s, notably in recent years. Ultrafine fibers or fibrous structures of different polymers can be created in the laboratory with a basic setup and have potential for industrial scale applications, which is perhaps partly responsible for the surge in interest in nanotechnology.

### 2.2. The Electrospinning Process

**Figure 3:**
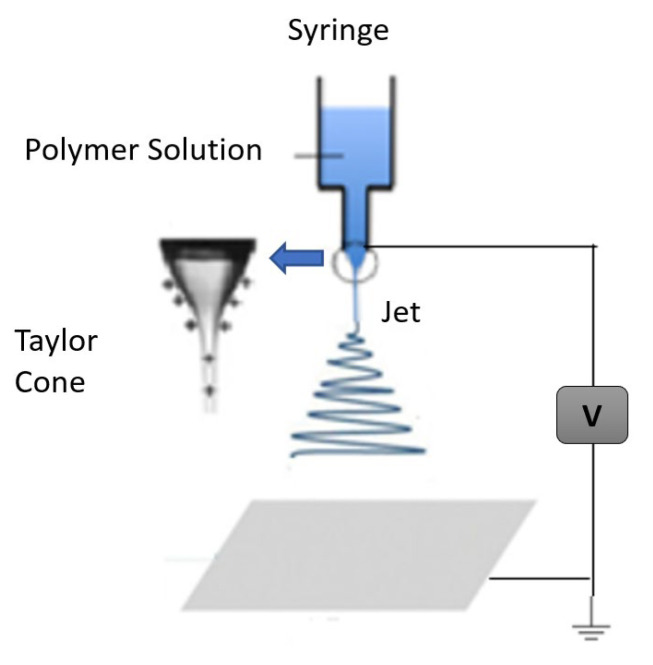
Diagrammatic illustration of electrospinning setup.

An electrospinnable polymer solution or melt must be able to carry a charge and have sufficient viscosity to stretch without disintegrating into droplets [34]. To execute the procedure, three main requirements are needed: high voltage supply, a capillary tube with a small needle, and a grounded gathering screen [35,36]. The capillary tube is filled with polymer melt or solution as the initial step in the processing stage. However, occasionally, due to the usage of solvents, the polymer may release noxious odors. Thus, an adequate amount of ventilation is required in order to carry out the electrospinning in a chamber. High voltage, usually between 1 to 30 kV, is applied to suspend droplets of the polymer solution in order to electrify the liquid surface. This causes the droplet to deform into a conical object known as a Taylor cone. The charged jet of solution is propelled from the Taylor cone’s tip as electric voltage approaches a critical value, because the electrostatic force overcomes the surface tension of the droplets. As the jet travels toward a collector, solvents solidify or evaporate in less than a tenth of a second, and, ultimately, tiny fibers are gathered into the form of a web [10,37]. There are three types of instabilities that the jet might undergo during this process, namely: Rayleigh instability, bending instability, and whipping instability [11,23,27]. A typical setup for electrospinning is shown in Figure 3.

The Taylor cone develops at the capillary tip when electrostatic forces are greater than the surface tension force, and the solution stream is ejected. The molecular weight of the polymer, chain entanglement, and solvent used during execution determines whether the jet will form a continuous fiber or scatter into droplets. Some studies have noted that smooth fibers are created when Berry’s number, $B_{e}=\eta c$, which is a measure of polymer’s intrinsic viscosity (η) and polymer concentration (c), is higher than a certain critical value $B_{e\text{\_}cr}$, which is characteristic of polymer [38]. A polymer solution’s specific viscosity is calculated using the ratio:

(1) $$\eta_{sp}=\frac{\eta_{o}-\eta_{s}}{\eta_{s}}$$

where $\eta_{s}$ is solvent’s viscosity and $\eta_{o}$ is the polymer solution’s zero shear viscosity at concentration (c). The intrinsic viscosity η of a polymer is observed as a linear extrapolation of specific viscosity $\eta_{sp}$ calculated for different concentrations to the concentration at c=0; intrinsic viscosity η can also correlated to molecular weight MW of a linear polymer by the Mark–Houwink equation:

(2) $$\eta =KMw^{\alpha }$$

where K is constant. Both K and α depend on polymer, temperature, and solvent.

Under the effect of the applied potential field, when excess charges flow to or from the liquid and on the liquid’s surface, anions and cations are not evenly distributed. Free surfaces of solution are always equipotential surfaces and have charges distributed in such a way that this maintains a zero-electric field inside the liquid.

**Figure 4:**
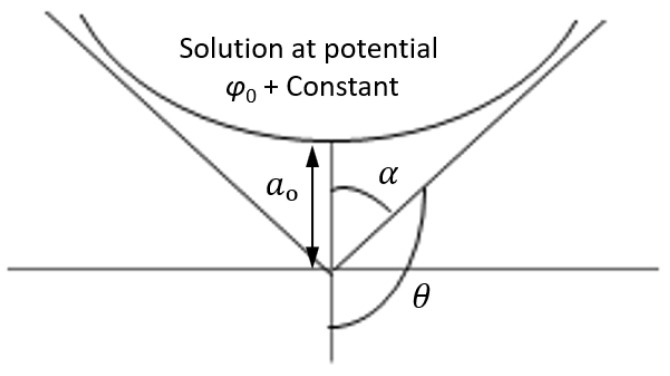
Axisymmetric “infinite” fluid under electrostatic force at a distance $a_{0}$ from an equipotential plane.

To demonstrate the self-similar nature of a solution corresponding to the Taylor cone, we suppose an axisymmetric liquid body which has the potential ($\varphi_{\mathrm{o;}}+\mathrm{const}$) at its tip and is kept at distance *a_o_* from an equipotential plane, Figure 4. The distribution of the electric potential is Φ=φ+const and is examined in the spherical coordinates R and *θ* and in cylindrical coordinates *ρ* and z. When the shape of the free surface is in equilibrium, surface tension force is balanced by electrical forces exerted on droplets. The solution should satisfy the Laplace equation, which makes it possible to find *Ψ*, as in Taylor (1964) [39].

(3) $$\Psi \left(\theta \right)=P_{½}\left(cos\theta \right)$$

where $P_{½}\left(cos\theta \right)$ is a Legender function of half order [40]. The free surface may have equipotential only when *θ* corresponds to zero of $P_{½}\left(cos\theta \right)$ in the range 0≤θ≤π, which is *θ*_o_ = 130.7° [39]. The droplet shown in Figure 4, is enveloped by a cone with the half angle at its tip equal to $\alpha =\alpha T=\pi -\theta_{o}=49.3{}^{\circ}$, which is a Taylor cone [27]. The shape of the droplet approaches the Taylor cone asymptotically as R→∞. (Note that Pantano et al., 1994 considered a finite drop attached to a tube [41].)

### 2.3. Electrospinning Techniques

There are, in general, different electrospinning techniques of nanofibers to respond to the specific demands of different industries and technologies. Table 2 presents the most important electrospinning techniques and their most important features.

### 2.4. Scalability of Electrospinning

A high-quality electrospinning process is greatly influenced by the physical design of the spinneret. The production rate of common single needle electrospinning equipment is approximately 0.01–0.1 g/h [50]. Generally, needleless and multi-needle electrospinning are approaches to improve the production of polymer nanofibers. In the multi needle spinning process, multiple needles are arranged in a certain pattern to increase the number of jets. However, the interference of electric fields and clog up of needles with a polymer solution constitutes a hindrance for industrial scale applications [51]. In needleless electrospinning, the polymer solution is self-energized to produce the capillary wave array as Taylor cones on a free liquid surface due to electrohydrodynamic instability. This technique increases the number of jets, overcomes the issue of clogging the nozzles, and improves the production of polymer nanofibers.

**Figure 5:**
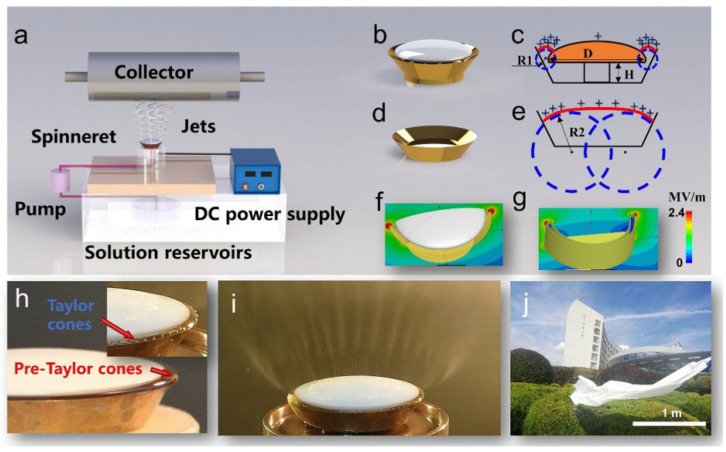
Mushroom-electrospinning process: (**a**) apparatus, (**b**) spinneret, (**c**) surface curvature and charge distribution on spinneret, (**d**) uncovered spinneret, (**e**) surface curvature and surface charge distribution of the uncovered spinneret. Concentrated electric field state of (**f**) the spinneret and (**g**) the uncovered spinneret. (**h**) Digital photograph of spinneret filling with solutions; the inset picture is photo of an annular array of Taylor cones under external electric fields. (**i**) Photo of multiple jets during electrospinning. (**j**) Resulting large-scale nanofiber membranes without any substrate via mushroom-electrospinning. Reproduced from [58] with the permission of Elsevier.

Needleless electrospinning is classified into rotating and stationary electrospinning. The rotating needleless setup consists of rotating discs [52], rotating cylinders [53], rotating spiral coils [54], and rotating cylinders as spinnerets, and stationery needleless consisting of bowl-shaped spinnerets [55], pyramid-shaped spinnerets [56], and metallic slit spinnerets [57] are most commonly used. During the process, auxiliary airflow, shear force, and other forces are developed to promote jet excitation and stretching. Due to the high excitation voltage, inadequate stability of free liquid, and difficult to control spatial movements of jets, high-quality electrospinning production volume is restricted. Jian Xiong et al. [58] developed stable annular pre-Taylor cones having high curvature which facilitate the formation of jets on a free liquid surface. Figure 5 shows an illustration of the mushroom electrospinning process. Mushroom-spinneret was developed to ensure steady movement of multiple jets. It is observed that critical excitation voltage was significantly reduced from 45 KV to 20 KV. Fabricated nanofiber membranes exhibit narrow distribution of diameters (CV ~ 10%), and spinnerets show the production capacity of 13.7 g/h.

### 2.5. Portable Electrospinning

The concept of in situ electrospinning was introduced for directly electrospinning fibers on the spot, for the directed purpose. This is mostly driven by applications in the medical field in wound dressing, where the efficacy of the in situ electrospun nanofibers is superior to that of stored ones [59]. As this technology is very much related to the biomedical field, more details will be given in the section dedicated to biomedical applications (Section 7.1).

## 3. Polymers Used in Electrospinning

The most prominent characteristic of electrospinning is its ability to process a huge variety of polymers to generate nanofibers for a variety of applications. Polymers can be categorized into synthetic, natural, or mixed polymers. Each category will be discussed in this section, with special significance placed on the electrospinning process.

### 3.1. Natural and Synthetic Polymers

Generally, electrospun nanofibers which are made of natural polymers replicate the physicochemical properties of the extracellular matrix. Natural polymers are composed of lipids, proteins, nucleic acids, and polysaccharides, etc. In the medical field, natural polymers showed superior biocompatibility and decreased immunogenicity in contrast to synthetic polymers. Natural polymers have an inherent affinity of closeness to cells because they have a unique protein arrangement, as in glycine and aspartic acid, which makes them a strong candidate for tissue engineering technology. Natural polymers consisting of silk fibroin, collagen, elastin, casein, cellulose acetate, gelatin, chitosan, chitin, fibrinogen, etc. are frequently reported for electrospinning. Tissue scaffolds based on natural polymer function better in clinical settings. Nevertheless, synthetic polymers are more advantageous than natural ones due to their superior mechanical properties, such as strength and viscoelasticity, and higher rate of degradation. In medical applications, synthetic polymers such as polylactide, polyglycolide, and poly (E-caprolactone) are frequently utilized as hydrophobic biodegradable polyesters [8]. Table 3 lists commonly used polymers in electrospinning process, their characterization techniques and applications.

### 3.2. Composite Polymers/Copolymers

It was demonstrated that it is feasible to combine the advantages of natural and synthetic polymers through using both of them in the electrospinning process. For example, various studies were conducted on combinations of polycaprolactone coated with gelatin, silk/polyethylene oxide blend, collagen/polycaprolactone—poly-L-lactic acid blend, hyaluronan and polycaprolactone, polycaprolactone–polylactic coated with collagen, starch/polycaprolactone blend, chitosan–polyethylene oxide, poly-L-lactic acid or poly(lactic-co-glycolic acid), or a mixture of natural polymers such as collagen and chondroitin sulphate, collagen and chitosan, collagen and elastin, collagen and PHBV, and gelatin with PHBV [86,87,88]. Copolymers can also be used in electrospinning to alter cellular affinity, morphology, mechanical characteristics, and various physical properties. For instance, the spinning blend of an ethylene-co-vinyl alcohol nanofibrous mat was strengthened by incorporation of glycolide. The effective compromise between decomposition speed and hydrophilicity was shown by a three-block copolymer made of polylactic acid, p-dioxanone, and polyethylene glycol.

## 4. Electrospinning Process Parameters

During the electrospinning process, the polymer solution is converted into nanofibers, which is influenced by various factors [36,89]:

- Parameters related to the solution, such as polymer molecular weight, polymer concentration, surface tension, conductivity, solvent volatility, and viscosity.
- Parameters related to processing such field strength, flow rate, tip-to-collector separation, applied voltage, placement and design of the needle tip, composition and geometry of the collector, and take-up velocity of the collector.
- Properties related to environmental factors including temperature, humidity, and pressure.

Changes in any parameter among these sets will definitely change the evolution of the electrospinning process and, thus, will change the characteristics of the resulting nanofibers. Therefore, it is important to investigate these parameters within their containing sets and assess respective effects on the process. In the following, we will discuss these factors.

### 4.1. Parameters Related to the Polymer Solution

Among the different variables affecting the outcome of the electrospinning process, the characteristics of the polymer solution have the main effect on the development polymer nanofibers with a broad range of sizes and morphologies. This can be categorized in the following.

#### 4.1.1. Concentration of the Polymer

For chain entanglement to take place, the polymer concentration should be at the optimum level: not too low, not too high. To produce continuous fibers, viscosity and surface tension have a prominent role for selecting the concentration of polymer. In the electrospinning process, the polymer solution with a low concentration affects the surface tension, which leads to synthetization of beads instead of fibers [90]. Additionally, when the polymer solution is highly concentrated, it is stopped at the capillary tip due to high viscosity, which disturbs the feed rate. Investigating the electrospinning of polyethylene oxide/water solutions consisting of different polyethylene oxide concentrations revealed that low viscosity levels of less than 800 centipoises allowed for the appearance of droplets, whereas at high levels of viscosity of more than 4000 centipoises the solution is too thick to be electrospun. In another study, it was reported that increasing the polystyrene concentration in tetrahydrofuran led to a smaller pore-size distribution while increasing fiber diameter. Combining them together, across a range of optimum concentrations, an increase in concentration led to larger fiber diameters [8,22,91].

#### 4.1.2. The Solvent

Choosing an appropriate solvent to prepare the polymer solution is an essential step in its spinability. The selected solvent is to have the right properties, such as appropriate evaporation rate, boiling point, and vapor pressure. In case of a binary solvent, molecular interactions, in terms of attraction or repulsion, entirely depend on solvent variety. As the polymer jet travels from the tip to the collector, solvent evaporation and phase separation take place simultaneously prior to the deposition of solid polymer fibers. This process is substantially influenced by the solvent evaporation rate [92]. The solvent vaporization rate is significantly influenced by the solvent vapor pressure [10]. Different solvents such as ethanol, hexafluoro isopropanol, chloroform, dichloromethane, and dimethylformamide are utilized for the electrospinning of synthetic polymers. Results confirm that physical characteristics of the polymer depend upon the selection of solvent. Natural solvents are expensive and also harmful to the environment. Furthermore, the trace amounts of organic solvents that remain in electrospun materials are detrimental for biological use, such as in tissue engineering and wound healing. Thus, prior to application, extra washing or purification is required [22]. According to Renker and Doshi, the creation of fibers without beads is facilitated by lowering surface tension of the polymer solution. Since surface tension is more dependent on composition of the solvent than on concentration of polymer, less surface tension of the solvent is not always favorable for electrospinning [10].

**Figure 6:**
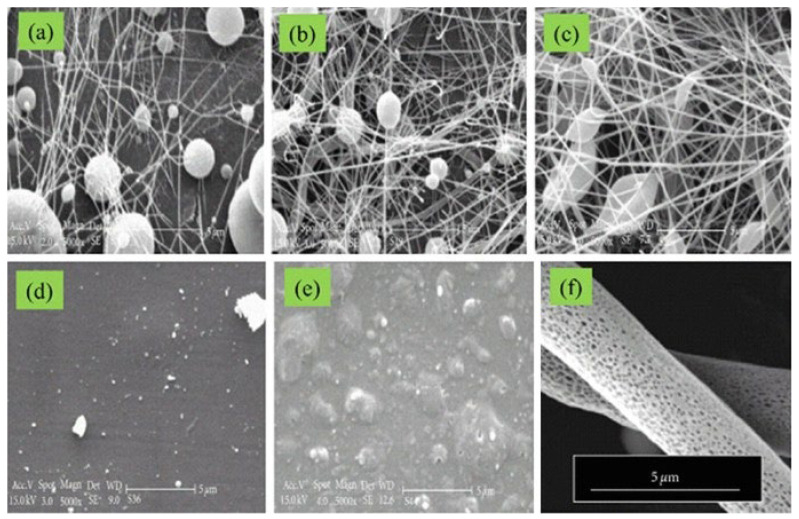
SEM images of 5% polycaprolactone solutions dissolved in various solvents: (**a**) glacial acetic acid, (**b**) 90% acetic acid, (**c**) methylene chloride/DMF = 4/1, (**d**) glacial formic acid, (**e**) and formic acid/acetone, along with (**f**) SEM images of polyvinyl butyral nanofibers prepared from 10 wt.% tetrahydrofuran/dimethyl sulfoxide (9/1 *v*/*v*). Reproduced from reference [92] with permission from Elsevier and Arabian Journal of Chemistry.

Morphology of fibers is also influenced by the rate of evaporation of the solvent. Rapid evaporation may cause the generation of flat fibers as opposed to the more typical round ones. Flat fibers actually develop when a little solvent is entrapped among fibers, causing the fiber to flatten out as the solvent evaporates [23]. Although it is highly influenced by processing conditions, utilizing a more volatile solvent is recommended among the alternatives [8,86].The selection of solvent influences fiber diameter, as depicted in Figure 6. Dimethylformamide solution, or a tetrahydrofuran and dimethylformamide solution blend, produces electrospun polyvinyl chloride fibers with a smaller diameter than pure tetrahydrofuran solution. Fiber porosity is also affected by solvent types as shown in Figure 6. Megelski et al. investigated structural characteristics of the electrospun polystyrene fibers made from solutions with different concentrations of dimethylformamide and tetrahydrofuran. High density pores were seen during the electrospinning of solutions containing 100% tetrahydrofuran (more volatile); enhancing fiber surface area by more than 20–40% depends upon the fiber diameter. However, using electrospinning solutions containing 100% dimethylformamide, smooth fibers with nearly no microtexture have been created (low volatility). Between these two limits, pore density decreased as a result of increased pore size and decreased pore depth [93].

**Figure 7:**
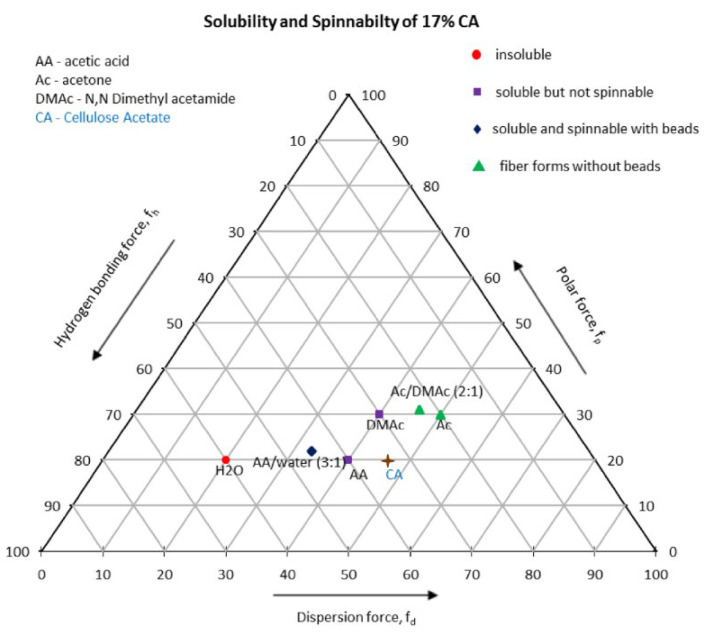
Teas chart to analyze the feasibility of solution formation and spinning with different solvents for cellulose acetate (CA). Reproduced from [94] with the permission of Springer Nature, Copyright, Indian Academy of Sciences.

The choice of a good solvent and preparation of a suitable solvent system for the desired polymer are necessary for the generation of electrospun polymer nanofibers. Solubility parameters are combined to aid in the search for the desired solvent system. In literature, ternary solubility diagrams, also known as Teas graphs, are widely used for the selection of solvent systems. Fractional cohesion parameters *f_h_*, *f_d_*, and *f_p_*, which were introduced by Teas, are derived from the hydrogen bonding component *δ_h_*, dispersion force component *δ_d_*, and polar force component *δ_p_* of Hansen parameters. Any solvent with specified Hansen parameters can be traced by plotting corresponding fractional parameters on a Teas graph. In one study, three solvent systems (acetic acid/water, acetone/Dimethylacetamide, and pure acetone) were used to prepare different cellulose acetate (CA) solutions with concentrations between 10 and 20 weight percent and also with different solvent concentrations. The solvents were analyzed by Hildebrand solubility parameter *δ*, and values for Hansen parameters were calculated for all solvents. The authors developed a Teas graph for the experiment, as shown in Figure 7 [94].

#### 4.1.3. Electrical Conductivity

In the electrospinning technique, the electrical conductivity of polymer melts or solution is an important factor. The type of polymer utilized, the solvent employed, and the presence of ionizable salts all influence in determining the conductivity of a polymer solution [22]. In comparison, conductive solutions have a higher charge density than less conducting solutions. Therefore, in the presence of an electric field, a high conductive fiber jet experiences higher tensile force when compared to a less conductive one. It was discovered that during spinning, raising the electrical conductivity of the solution causes a sizable decline in the diameter of nanofiber. By contrast, with low conductivity, the electric field is unable to provide enough jet elongation to generate uniform fibers, leading to the appearance of beads. According to Hayati et al., high conductive solutions are highly unstable in the existence of a robust electric field, which may cause significant bending instability and a wide range of diameter distribution [95]. Conversely, semiconducting and insulating fluids, such as paraffinic oil, were able to create fibers that were comparatively stable. Additionally, it was discovered that the fiber jet’s radius is inversely proportional to the cubic root of the solution’s electrical conductivity. In comparison to synthetic polymers, natural polymers such as gelatin, that are polyelectrolytes by nature, have less ability to form fibers. Ions undergo additional stress during the electrospinning process when they are exposed to an electric field, as their carrying charge capacity is increased. Salt addition results in thinner, more uniform fibers, and it also reduces the development of beads [10,92]. This phenomenon is caused by a rise in charge density, which enables the jet polymer to be subjected to greater elongation and dielectric stresses, leading to the formation of thinner fibers [86].

#### 4.1.4. Viscosity

**Figure 8:**
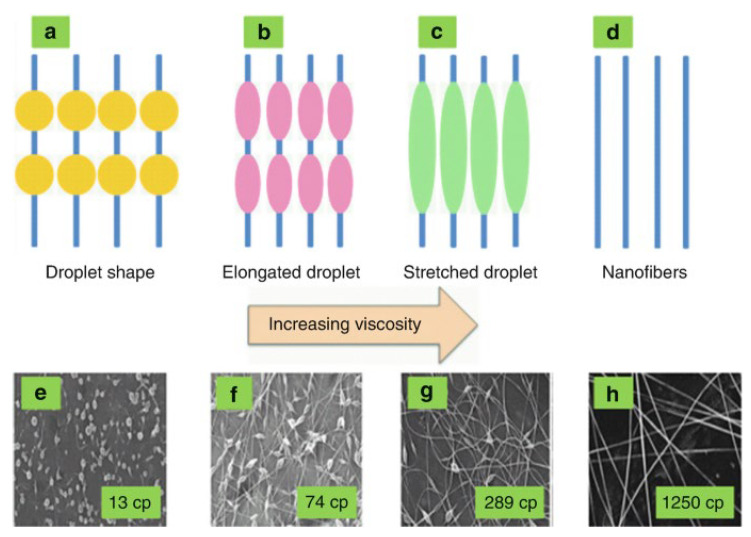
Modification in morphology of electrospun nanofibers of polyethylene oxide with viscosity: (**a**–**d**) schematic and (**e**–**h**) SEM micrographs. Reproduced from [92] with permission from Elsevier and Arabian Journal of Chemistry.

Most of the research indicates that viscosity is a prominent factor in analyzing diameter and morphology of the fibers. Enhancing the viscosity by molecular weight or by an increase in the polymer’s concentration increases the production of polymer fibers with a larger diameter, while the probability of bead generation is reduced, and fibers are more uniform in appearance. It was demonstrated that at very low viscosities, due to the deficiency of chain entanglement, there is no formation of any continuous fiber, and the fiber jet breaks into droplets. On the other hand, when a polymer solution has very high viscosity, it is difficult to shoot a jet from the solution, because the polymer flow stops at the needle’s tip. Viscosity of the solution is significantly correlated to the concentration of polymer. The relation between viscosity, concentration, and morphology of electrospun fibers has been examined by investigating polylactic-co-glycolic acid, polyethylene oxide, polyvinyl alcohol, poly-methyl methacrylate, and poly-l-lactic acid. Viscous solutions exhibit a longer duration for stress relaxation in the electrospinning process. Furthermore, by raising viscosity by concentration of the solution, the bigger and much more uniform fibers are produced. Viscosity is essential in analyzing the range of concentrations at which continuous fibers may be produced. Solutions having less viscosity and surface tension are prominent indicators for the generation of beaded fibers. Continuous nanofibrous structures were created while the concentration was above the optimal level, and morphology was influenced by the solution’s concentration [7,10,96,97]. In electrospinning, each polymer has an optimal viscosity which has significant influence on morphology of the resulting polymer nanofiber, as shown in Figure 8.

#### 4.1.5. Molecular Weight

Polymer molecular weight is another vital parameter related to the solution which influences the morphology of the polymer nanofiber. The rheological properties, such as viscosity and surface tension, conductivity, and dielectric strength, are significantly influenced by molecular weight [98]. Therefore, large molecular weight polymers are typically employed to manufacture fibers because they offer the appropriate viscosity. Furthermore, Casper et al. discovered that raising the polymer’s molecular weight causes variations in the fiber’s morphology, such as reduction in the number of beads and irregular form, while also enlarging pore size. As a result, it became possible to create a mesh with more consistent biophysical properties [86,99]. Low molecular weight solutions typically result in beads rather than fibers. In other words, electro-spraying rather than electrospinning occurs in solutions having less molecular weight. However, the increasing molecular weight causes the fibers’ average diameter to increase [22]. In the electrospinning process, chain entanglement plays a significant role, for example, when high molecular weight poly-L-lactic acid is utilized in spite of the low polymer concentration. It was able to provide enough chain entanglement and viscosity for the creation of uniform fibers. Moreover, the effects of surface tension are minimized, which is significant for the creation of beads.

It has been investigated that chain entanglement is more significant than high molecular weight when it comes to manufacturing uniform nanofibers. It is noted that the formation of nanofibers will take place if interactions between polymer molecules are sufficient to qualify for chain entanglement. For instance, the electrospinning behavior of polymers having strong quadruple hydrogen-bonding capacity resembles that of large molecular weight nonfunctional polymers. Utilizing the same idea, nonwoven membranes are generated by oligomerized phospholipids from lecithin solutions, utilizing electrospinning technique. However, despite the fact that chain entanglement of polymer to produce fibers is a significant property, solution viscosity is a much more general characteristic, as ceramic substrates can still be electrospun despite having a low molecular weight [9].

### 4.2. Parameters Related Electrospinning Equipment

#### 4.2.1. Applied Voltage

**Figure 9:**
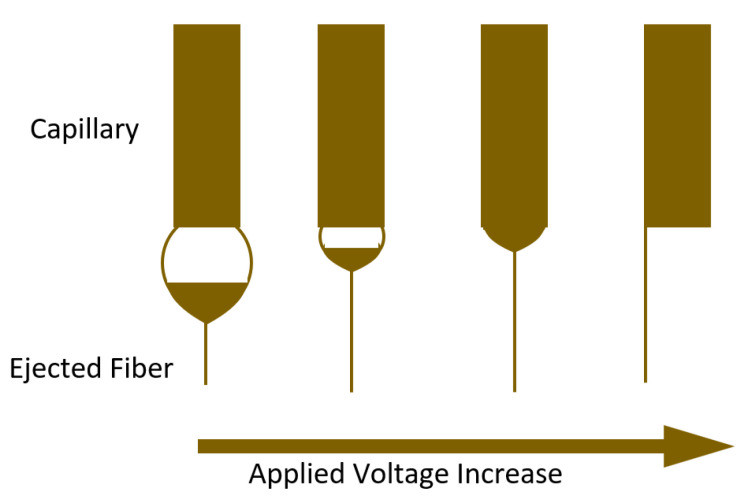
Influence voltage on the Taylor cone creation. At low voltage, a pendant droplet (white) is produced at the capillary tip while a Taylor cone (dark blue) is produced at the pendant droplet tip. By enhancing voltage, a Taylor cone is produced at the capillary tip, and then further voltage results in the ejection of the fiber jet through the capillary.

The influence of voltage on the size and morphology of electrospun polymer nanofibers has been subjected to significant controversy. It has been suggested that thicker fibers are produced by higher voltage, due to many exiting polymers. Others, however, have asserted that thinner fibers are produced as voltage increases because electrostatic repulsion forces are applied to the polymer jet. However, in the majority of situations, the rapid solvent evaporation and lower fiber diameter are caused by the higher enforced electric forces in the jet. Therefore, at high voltages, there is more chance of bead production. According to Larrondo and Manley, the diameter of the fiber is reduced approximately to half when the applied electric field is doubled. Deitzel et al. examined polyethylene oxide/water and observed that by enhancing voltage, the shape of the surface is altered where the Taylor cone and jet fiber formed, as shown in Figure 9 [87] [92].

#### 4.2.2. Feed Rate

Polymer solution delivery speed and jet intensity are influenced by polymer flow rate within a syringe, which influences the diameter and morphology of fibers. Electric field intensity, gravity, and pumping pressure all have an impact on how quickly the polymer solution flows during the electrospinning process. Increasing the polymer solution feed rate resulted in a rise in fiber diameter, while a slower flow rate can be used to create thinner fibers. The slower feed rate is preferable, as the solvent has adequate time to evaporate. Larger flow rates, however, result in the development of beaded fibers because the solvent does not have enough time to evaporate before approaching to collector plate. Zuo et al. reported similar outcomes as well [8,10]. Porosity and shape of fibers are also influenced by the flow rate. Megelski et al. examined the influence of flow rate on the structure of electrospun fibers of polystyrene and tetrahydrofuran: both pore size and fiber diameter increased by enhancing flow rate. At a high flow rate, fibers were unable to completely dry before reaching the collector, which causes the considerable bead flaws [86]. Additionally, incomplete drying of fibers produced flat or ribbon-like fibers, as opposed to fibers having a round cross section [92].

#### 4.2.3. Distance between Tip and Collector

The distance between spinneret and collector affects morphology and fiber diameter, as well. For fibers to have sufficient time to dry before they reach the collector, there must be an optimum distance. If not, beads are produced at either too near or too far a distance. Distance can determine whether electrospray or electrospinning will ultimately occur. Additionally, it is noted that increasing distance results in more round fibers while decreasing it produces more flat ones [82]. Doshi and Renker observed that fiber diameter decreases with increasing distance from the Taylor cone. Additionally, Jaeger et al. investigated how the diameter of electrospun fibers made from polymer solutions of polyethylene oxide/water changed with distance from the Taylor cone. According to one study, increasing the spacing from 1 to 3.5 cm resulted in a reduction of nearly two-folds in fiber diameter, from 19 μm to 9 μm [87]. Short distances will result in flat or quasi-ribbon fiber and a beaded structure regardless of concentration of the polymer solution [86]. This is related to the polymer fiber not being adequately dried before entering the collector [92]. Consideration of the optimum distance is also influenced by a lot of parameters, including the nature of the solvent. Therefore, for high volatile solvents, short distances were employed, whereas for low volatility solvents, such as water, larger distances are needed for sufficient evaporation. For fibers to have adequate time to dry before approaching the collector and producing uniform, bead-free fibers, a minimum distance of 8 to 15 cm is required [22].

### 4.3. Parameters Related to Environment

The quality of electrospun nanofibers is significantly influenced by environmental conditions, including temperature, air flow velocity, and humidity, in addition to electrospinning equipment and substrate parameters. Although electrospinning normally proceeds at room temperature, increasing the temperature from 25 °C to 60 °C results in smaller fiber diameters, which lowers viscosity. Temperature and viscosity have an inverse relationship [90]. It was shown that by enhancing humidity, tiny spherical pores begin to develop on the surface, and further raising the humidity will cause pores to gradually connect to each other. Extremely low humidity also presents a challenge, since the rate of solvent evaporation will increase and the solvent will dry out more quickly. However, greater humidity can result in the discharge of an electrospun solution. A larger fiber diameter is attained due to air flow above the injection needle that will cause an increase in the rate of evaporation through convection [10,86,99]. Table 4 provides a concise overview of parameters with their effects on electrospun nanofiber morphology.

## 5. Properties of Polymer Nanofibers

### 5.1. Mechanical Properties

Advanced nanomaterials and microstructural components are integrated with the electrospun nanofibers for the vast variety of applications in biomedical, textile, airborne, reinforcing elements in composite materials, filter media, structure, nano-sensors, etc. During their service lives, they are frequently exposed to stress and strain from the surroundings. The utilization of polymer nanofibers in such wide applications will be discussed at the end of this work. Such wide applications require that EPNF possesses the right mechanical properties for the desired functions. The mechanical behavior of the nanofibers includes the overall deformation under static and dynamic responses, friction, and contacts in polymer nanofibrous networks [100,101]. To determine the mechanical behavior of the EPNF structure, it is essential to examine characteristics of a single electrospun nanofiber. Generally, electrospun nanofibers are too thin and fragile, thus, during electrospinning process, they are accumulated on a nonwoven bundle. Therefore, determining the mechanical behavior of a single electrospun nanofiber is a challenging process that requires overcoming many obstacles using a sophisticated set of instruments. Among these instruments are the following [100,102,103,104,105]:

1. A manipulation system that precisely isolates, aligns, and grasps a single nanofiber on a frame without slipping or damaging.
2. A proper monitoring system to verify that nanofibers are not harmed by characterization tools such as scanning electron microscopes or transmission electron microscopes.
3. A sensitive force transducer having a range of nano- to micro-Newton range (n/μN range) resolution that can measure applied force in the n/μN range.
4. An actuator that is capable to load nanofibers until fracture, with high resolution (load unit: μN).

There were numerous attempts to investigate the mechanical behavior of electrospun nanofibers based on tensile tests on nanofibrous mats utilizing the universal testing machine [106,107]. The tension test entirely depends on nanofiber diameters, alignment, and entanglements inside nanofibrous mats. Therefore, this technique cannot be considered appropriate [108,109]. In order to address this problem, numerous alternative methods for the mechanical characterization of continuous nanofibers were presented [100]. Atomic force microscopy (AFM), which includes both force and distance sensors and can be operated in air, liquids, and vacuum, has become one of the generic characterization tools. However, AFM is just the primary tool for other measurement methods. Most of these methods rely on fibers’ tensile, bending, stretching, nanoindentation, and shear modulation frequency [100,110,111,112].

#### 5.1.1. The Tensile Test Method

In the tensile test method, the polymer nanofiber is primarily used to withstand axial loads. Tensile tests [103,113] are most appropriate to examine the mechanical behavior of individual polymer electrospun nanofibers. The tensile test system includes a specimen elongation measurement tool, a load, and an actuator. Tensile tests are carried out on nano-tensile/micro-tensile equipment or atomic force microscopy-based system to examine the property of interest, i.e., Young’s modulus, tensile strength, yield stress, and strain at fracture. These tests entail determining the applied load and fiber elongation of a known cross-sectional area [101].

#### 5.1.2. Atomic Force Microscopy Method for Tension Test

**Figure 10:**
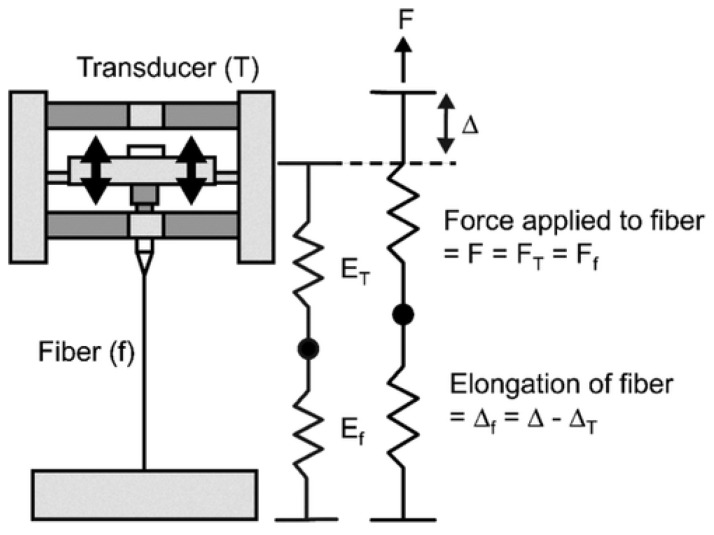
Schematic of microfiber–transducer configuration, Reproduced form [118] with permission of AIP Publishing.

In this method, normally, one end of the polymer nanofiber is tied on a substrate with an adhesive or electron beam-induced deposition technique, which acts as a pulling component. The second end is fastened to an atomic force microscope (AFM) tip which serves as the force detecting element [114,115]. A microscopic tensile force is applied by the movement of the AFM tip, and the stress–strain behavior is examined on the length of the nanofiber, which is simultaneously captured by scanning electron microscope [116] or optical microscope [117]. For tensile testing of micro- or nano-scaled fiber bundles, Tan and Lim, in 2004, developed an AFM-based nanoindentation device, wherein the microfiber was fastened between the tip and base of the nanoindenter, and the fiber was stretched by the stepper motor of the AFM system [118]. Load and elongation are calculated by a fiber-transducer series configuration, as shown in Figure 10.

**Figure 11:**
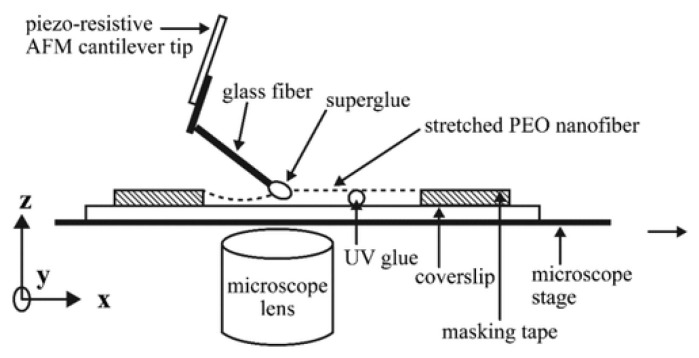
Diagram of single PEO nanofiber tensile test utilizing piezoresistive AFM tip. Reprinted from [114] with permission from AIP Publishing.

Later on, Tan et al. used a different technique to calculate the tensile properties of single electrospun polyethylene oxide nanofibers [114]. For higher accuracy, flexible cantilever arms were equipped with resistive strain gauges. A piezoresistive cantilever tip connected to multimeter, resistance varying linearly as a result of cantilever tip’s deflection, and this change in resistance can be easily translated into load. Tensile testing of polyethylene oxide nanofiber by piezoresistive AFM tip is shown in Figure 11. Young’s modulus of polyethylene oxide (PEO) nanofiber having a diameter of 700 nm was calculated to be 45 MPa.

**Figure 12:**
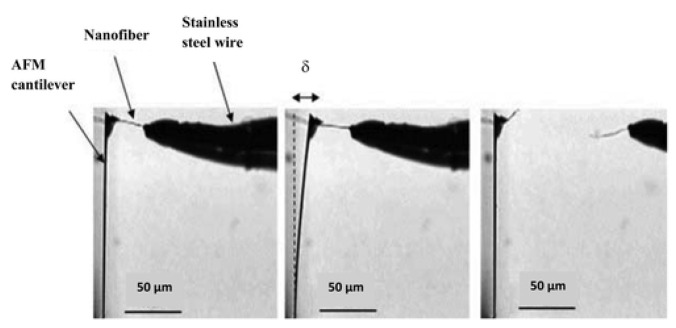
Tensile test of single-electrospun nylon-6,6 nanofiber up to fracture. Reprinted from [115] with permission from John Wiley and Sons.

Tensile behavior of electrospun nylon-66 nanofibers with an average diameter of 550 nm was analyzed. A stainless-steel wire was attached to one end of nylon-6,6 nanofiber, while the other end was connected to the cantilever tip which exerts force, as shown in Figure 12. An optical microscope was used to track cantilever deflection and elongation in the nanofiber. Young’s modulus was calculated from the generated stress–strain curve.

#### 5.1.3. Microelectromechanical System

**Figure 13:**
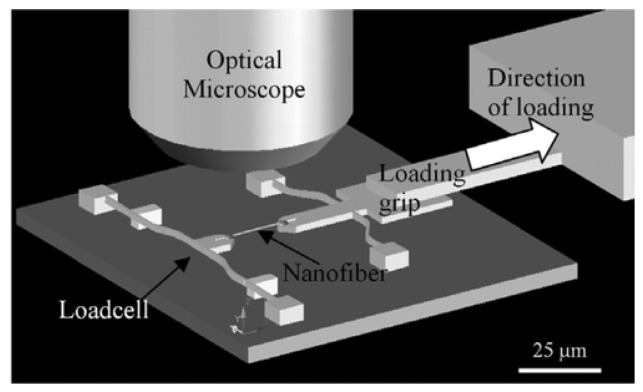
Microtensile testing platform for determining the mechanical characteristics’ single-electrospun nanofiber. Reprinted from [120] with permission from AIP Publishing.

To examine the load displacement relationship for a single polymer nanofiber, microelectromechanical systems equipment is designed with on-chip leaf-spring load cell and actuators with grip for fiber specimen mounting [119,120,121]. High manipulation accuracy is necessary to position the nanofiber between grasping components while being observed through an optical microscope [114,122] or scanning electron microscope [123]. Although the MEMS force sensor seems to be precise, using it requires in situ SEM or necessitates that the sensor is glued to fiber after being installed onto a MEMS testing stand [124]. Using MEMS-based equipment with on-chip leaf-spring load cell and hold specimens by piezoelectric actuator, Naraghi et al. applied three nominal strain rates of 2.5 × 10^−4^, 2.5 × 10^−3^, and 2.5 × 10^−2^ (S^−1^) on samples and observed behavior under optical microscope at 500 times magnification. Electrospun polyacrylonitrile (PAN) nanofibers having 300–600 nm diameters and lengths of 12 μm were investigated for mechanical deformation. PAN nanofibers were put on grips by a micromanipulator and connected with epoxy adhesive, as shown in Figure 13. From optical images, displacement of the fiber grips and the load cell’s deflection were simultaneously measured. Deflection of load cell and fiber grip’s displacement were calculated synchronously by optical images.

**Figure 14:**
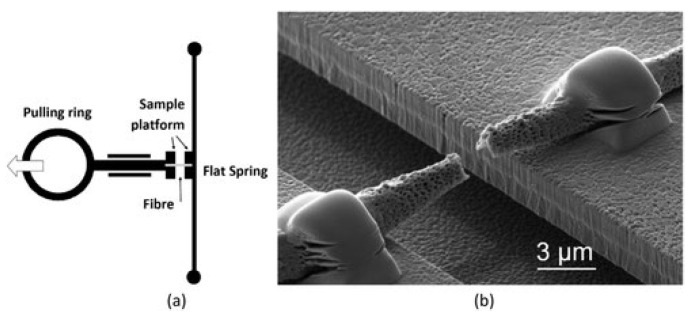
(**a**) MEMS-based tensile testing setup, and (**b**) SEM image of single nanofiber after tensile test. Reprinted from [123] with permission from John Wiley and Sons.

Mechanical behavior of poly (L-lactic acid) nanofibers having diameters of a 150 nm to 2μm range were investigated using a MEMS device [123]. The MEMS device has two movable elements which are cantilevers for bending and pulling. The pulling cantilever contains a pulling ring and a specimen stand which moves linearly under the guidance of two rails connected to a silicon chip. The bending cantilever consists of a stand for the sample and a silicon bar, attached at both ends to the silicon chip that serves as a flat spring. The fiber, which is under experimentation, is fastened by two sample stands in such a way that pulling and bending cantilevers are connected by the fiber, as shown in Figure 14. The flat spring exerts force on the fiber as the pulling cantilever moves. The force can be determined from the flat spring’s displacement on the assumption of linear elastic response of the silicon bar. By assuming linearly elastic behavior of the silicon bar, the force can be determined from displacement of the flat spring. Variations in displacements of the two specimen platforms can be used to calculate the fiber’s strain.

#### 5.1.4. Nano Indentation Method

**Figure 15:**
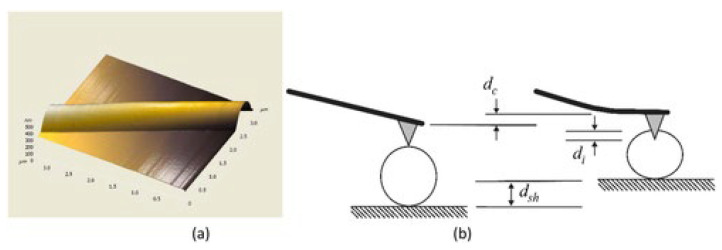
(**a**) Indentation test of a single nanofiber on a solid silicon. (**b**) A schematic explaining the deformation process in AFM measurement: before deformation (left) and after deformation (right). Reprinted from [113,127] with permission from John Wiley and Sons.

To determine the mechanical behavior of nanofibers, nanoindentation might be the most convenient technique to process, as a specimen is prepared by simply depositing fiber on a flat and hard substrate having adequate adherence to the substrate. Normal force is exerted by the AFM tip to produce a slight indentation on the nanofiber surface, as shown in Figure 15. After indentation, Young’s modulus can be calculated by probing localized curvature generated on the fiber’s surface [100].

The elastic modulus of electrospun nanofiber can be determined by fitting data with proper indentation models [125]. Nanoindentation is an indirect technique in which fiber undergoes localized deformation, used to predict the elastic modulus of fiber which depends on applying load and contact radius of the tip of the indenter [126]. Despite being one of the easy methods for characterizing a nanofiber’s mechanical properties, nanoindentation involves many variables that must be taken with some uncertainties [100]. Furthermore, such local tests do not reveal the dominant deformation mode, and failure of nanofibers in the axial stretching is expected [119].

#### 5.1.5. Shear Modulation Force Microscopy Method

**Figure 16:**
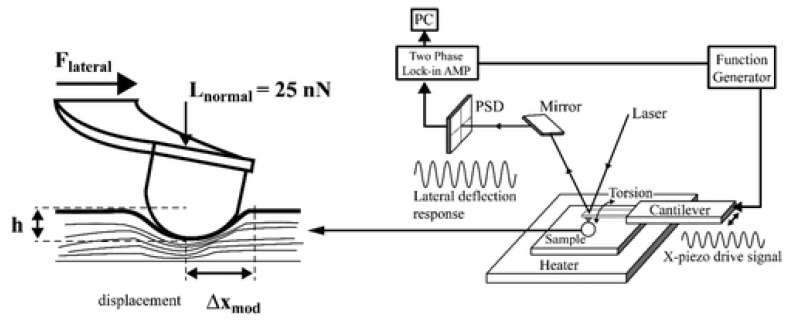
A schematic of shear modulation force microscopy. Reproduced from [29] with permission of the American Chemical Society.

In this method, the fiber bundle is suspended across the spacing of the grating, where slight oscillation is induced by the AFM tip, which is parallel to the specimen axis results in deformation, as shown in Figure 16.

Young’s modulus of the fiber is determined from the force displacement curve of the AFM probe, which is based on lateral deflection and Hertz model utilizing the following formula [29,40,128]:

(4) $$E=2\left(1+v\right)G$$

where *E* is elastic’s modulus, *G* is Young’s modulus, and *v* is Poisson’s ratio. This technique elaborates on the approximation of mechanical characteristics of individual fibers with higher sensitivity to the surface properties of nanofiber.

#### 5.1.6. Bending Test Method

An atomic force microscope paves way to investigate the mechanical behavior of single-electrospun nanofiber by its capability to exert forces in the nano-Newton and pico-Newton range on the surface of nanofibers and detect deflection of the cantilever in the Angstrom range [100,101,129]. Here, based on AFM technique, both two-point and three-point bending experiments are reviewed.

##### Two Point Bending Test Method

**Figure 17:**
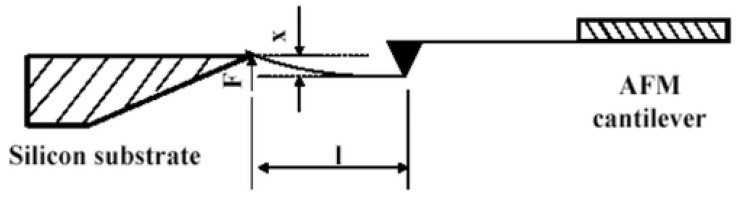
Schematic of shear modulation force microscopy. Reprinted from [29] with permission from AIP Publishing.

A two point bending experiment was performed on single electrospun polyacrylonitrile nanofiber by Gu et al. [104]. One end of the AFM cantilever is attached with single electrospun nanofiber by epoxy, and the second end was freely packed by the substrate edge, as shown in Figure 17. An optical microscope was utilized to analyze fiber’s displacement. Displacement and spring constant of the cantilever can be used to measure force. Young’s modulus $E_{Y}$ of nanofiber can be determined from the following formula:

(5) $$E_{Y}=\frac{4}{3\pi }+\frac{F}{x}+\frac{l^{3}}{r^{4}}$$

where *l* is the length of fiber, *r* is radius of fiber, and F/x is the product of the slope of the force-displacement curve fitting line and the cantilever’s spring constant.

##### Three-Point Bending Test Method

**Figure 18:**
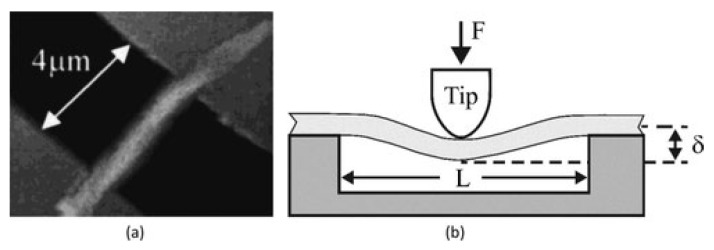
(**a**) Single-electrospun nanofiber suspended over etched grooves of silicon wafer, and (**b**) schematic of nanofiber with mid-span deflected by AFM tip. Reprinted from [131] with permission from AIP Publishing.

In the three-point bending technique, a single nanofiber is deposited over an etched groove (normally in glass, silicone, or polydimethylsiloxane), as can be seen in Figure 18a [130,131,132]. A sample of the nanofiber is clamped at both ends by an adhesive. The AFM tip exerts a transverse force at the center of a suspended nanofiber (Figure 18b). The elastic modulus can be calculated from the beam bending theory [133]. The nanofiber is supposed to act as an elastic beam fixed at both ends, which undergoes pure bending deformation (i.e., insignificant shear deformation). The elastic modulus can be represented as:

(6) $$E_{e}=\frac{PL^{3}}{192\partial I}$$

where *P* is the applied force, *L* is the clamped length, ∂ is the beam deflection at midspan, and I is the second moment of area of the beam [131,134].

Utilizing this method, the elastic modulus was calculated for two types of electrospun fibers, which are titanium dioxide (anatase) and polyvinylpyrrolidone/titanium dioxide nanocomposite with average fiber diameters of 53 and 109 nm, respectively [135]. Young’s modulus for TiO_2_ was found to be 0.9 GPa, and for PVP/TiO_2_ nanocomposite fibers, it was 75.6 GPa. This method has been utilized to examine the elastic moduli of other electrospun nanofibers and nanocomposites [136,137,138,139].

**Figure 19:**
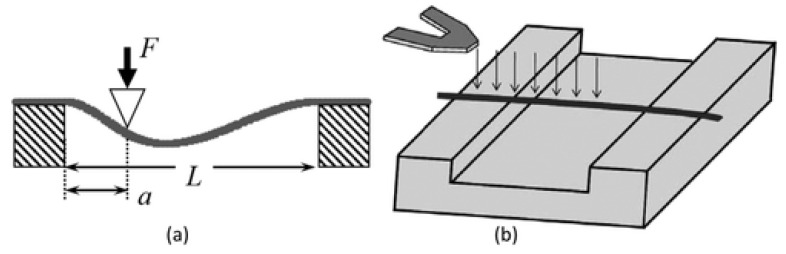
(**a**) A model for the fiber clamped to supports that are apart by distance L under deformation exerted by vertical force F at a distance “*a*” from one end of fiber. (**b**) Schematic of three-point bending test for single electrospun nanofiber. Reproduced from: (**a**) [140] with permission © 2005, American Chemical Society; (**b**) [141] with permission of Elsevier.

Based on the three-point bending concept, the multipoint bending technique was presented by Guhados et al. [140]. In this bending technique, the cantilever applied a known force at various points along a suspended nanofiber, and deflection of the fiber was calculated for each point, as shown in Figure 19a.

In the multi-point bend technique, to prevent damage to the nanofiber surface, the AFM cantilever tip can be replaced by a tipless cantilever [141]. In this technique, deflection versus piezo-displacement curves are measured at various points along fiber suspended across channel (Figure 19b). The primary benefit of the multipoint bending test approach is that it does not involve exactly locating the center point of the nanofiber [141].

#### 5.1.7. Stretching Method

**Figure 20:**
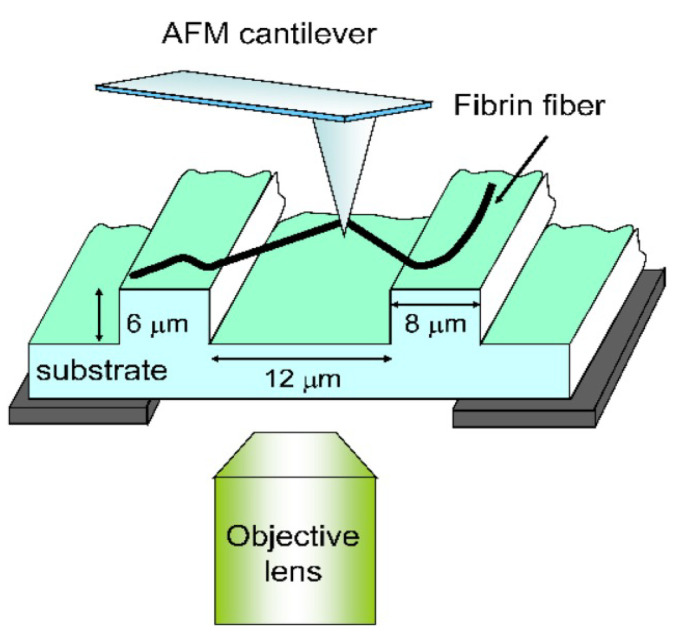
Schematic of AFM/optical microscopic apparatus setup. Reproduced from [142] with the permission of Elsevier.

Guthold et al. examined the mechanical characteristics of a single electrospun fibrinogen fiber by combining an AFM and fluorescence microscope in aqueous buffer [142]. Fibers having 208 nm average diameter were hanged on 12 μm wide ranging grooves in a transparent and striated substrate. Fibers were stretched laterally by AFM, and the stretching process was observed by a fluorescence microscope which was adjusted below the specimen, as in Figure 20. 

Mechanical properties of a single electrospun fibrinogen were evaluated at ambient and dry conditions utilizing the same methodology. Results indicate that nanofibers having a 30 to 200 nm diameter range stretched up to 74% elongation before fracture at a stress of 2.1 GPa. Fibers showed elastic behavior up to 15% elongation [143].

Another study utilized the same technique, to examine mechanical characteristics of individual electrospun collagen type I nanofibers with an average diameter of 160 to 783 nm. It was noted that the nanofibers’ strain softening, peak stress, and modulus are highly dependent on the fiber dimensions. The reported results reveal that electrospun collagen undergoes severe strain softening [144].

### 5.2. Chemical Properties

Fourier-transform infrared spectroscopy, nuclear magnetic resonance, circular dichroism, differential scanning calorimetry, X-ray scattering, and X-ray diffraction are commonly used to characterize the chemical composition of nanofibers. The vibrational spectroscopic technique is used for molecule structure analysis. This approach assists in determining chemical reactions between ingredients of polymers in case of polymer blends [23]. Raman spectroscopy is utilized to examine the structural characteristics of carbonaceous polymers. This was used by Sadrjahani et al. to assess the molecular orientation of electrospun polyacrylonitrile nanofibers [145]. A chain alignment parameter of 0.25 was calculated for nanofibers which accumulated at a 59.5 m/min take-up velocity. Surface chemical characteristics of polymer nanofibers can be analyzed by making use of their hydrophilicity. The latter is calculated by water contact angle, and it is examined by attenuated total reflectance Fourier transform infrared and X-ray photoelectron spectroscopy. Element detection by X-ray photoelectron spectroscopy is possible up to depth of 100 Å [146]. This technique is used to check the shell within core-shell structure of electrospun nanofibers, not to form a blend or react chemically with core [147]. Supermolecular structures, which are referred to as macromolecular configurations in polymer nanofibers, can be examined by small angle X-ray scattering, wide angle X-ray diffraction and differential scanning calorimetry [148]. The crystalline phase and crystal type are identified by wide angle X-ray diffraction, while small angle X-ray scattering is used to examine the lamellar structure of semicrystalline polymers. Other equipment is not used commonly due to complications in interpreting its pattern.

### 5.3. Thermal Properties

Thermal characterizations of electrospun nanofibers, such as melting and crystallization processes, can be determined by differential scanning calorimetry. To carry out this procedure, electrospun mats weighing around 10 mg are placed in a sealed aluminum pan. To preserve ambient pressure and promote evaporation of leftover solvents, pan covers are equipped with holes. Then, the specimens’ temperature is raised from 30 °C to 300 °C while maintaining a heating rate of usually 10 °C/min and a steady flow of dry nitrogen. The following equation determines the percent crystallinity ($\chi_{c}$) [149]:

(7) $$\chi_{c}\left(\%\right)=\frac{\Delta H_{f}-\Delta H_{c}}{\Delta H^{{}^{\circ}}{}_{f}}\times 100$$

where the enthalpy of crystallization, $\Delta H_{c}$, and the melting enthalpy, $\Delta H_{f}$, are acquired from differential scanning calorimetry traces, and the heat of fusion $\Delta H^{{}^{\circ}}{}_{f}$ is a thermal property of a perfectly crystalline substance [149,150]. In comparison to neat polymer, crystallinity of nylon-6,6 electrospun nanofiber is high, which could be a result of shear stress applied on the polymer jet during the electrospinning process [151]. Peresin et al. made a similar observation and noted that electrospinning significantly increased the degree of crystallinity of polyvinyl alcohol and raised 2 °C in melting temperature. These results are attributed to the orientation and improved crystallization of polymer chains in individual polyvinyl alcohol fibers which undergo extreme shear stress during electrospinning process [152].

### 5.4. Electrical Properties

**Figure 21:**
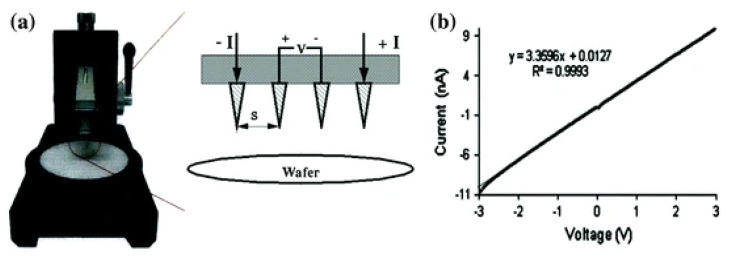
(**a**) Four-point probe set-up and (**b**) I–V curve at a voltage in range from −3 to +3 V for electrospun PLLA/PANI nanofibers. Reproduced from [155,156] with the permission of Elsevier.

To analyze the electrical properties of nonwoven mats and single fibers, electrodes are pre-patterned on a substratum or vaporized on top of electrospun polymer materials [153]. To measure electrical conductivity readings from current voltage curves, generally four-point probe [154] or two-point probe [155] measurements are utilized. When using a four-point probe, a material of unknown resistance is contacted by four evenly spaced probes, as in Figure 21. Measurements based on this technique assume a thin film instead of a porous fiber network. As a result, measured conductivity measurements can be lower than the actual measurement for bulk films. Another source of uncertainty for electrical conductivity is the pins’ depth (height) of penetration into fiber mats [156]. Penetration of pins into a polymer fiber mat could be the cause of uncertainty in determining the electrical conductivity.

**Figure 22:**
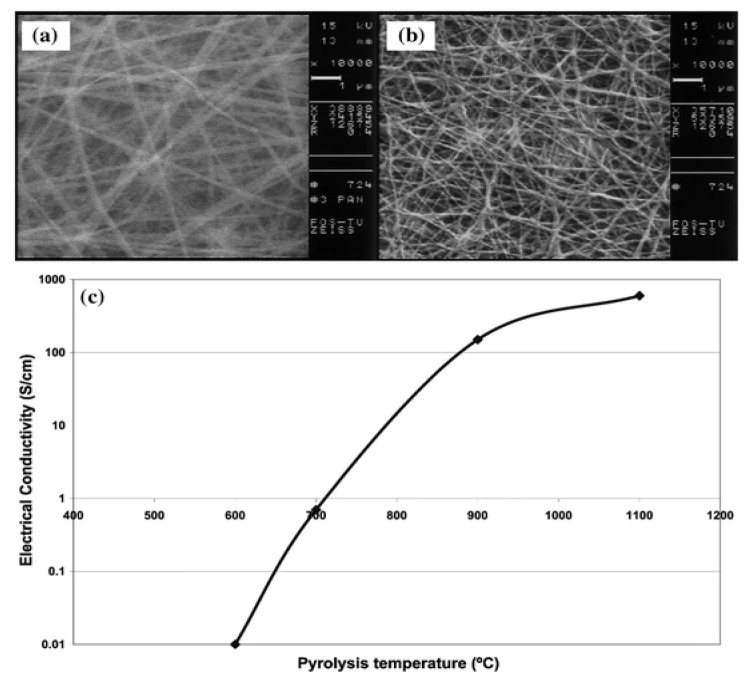
SEM images of (**a**) PAN, (**b**) carbon nanofibers without a gold coating, and (**c**) pyrolysis temperature versus conductivity. Reproduced from [157] with the permission of John Wiley and Sons.

Agend et al. utilized scanning electron microscope images of carbonized polyacrylonitrile nanofibers to evaluate its electrical properties, using a gold-coating technique on some samples [157]. It was possible to obtain the electrical properties of carbonized polyacrylonitrile nanofibers, as demonstrated in Figure 22a,b. It shows a significant increase in conductivity with the increase in pyrolysis temperature during measurement of conductivity behavior by four-point probe (Figure 22c).

**Figure 23:**
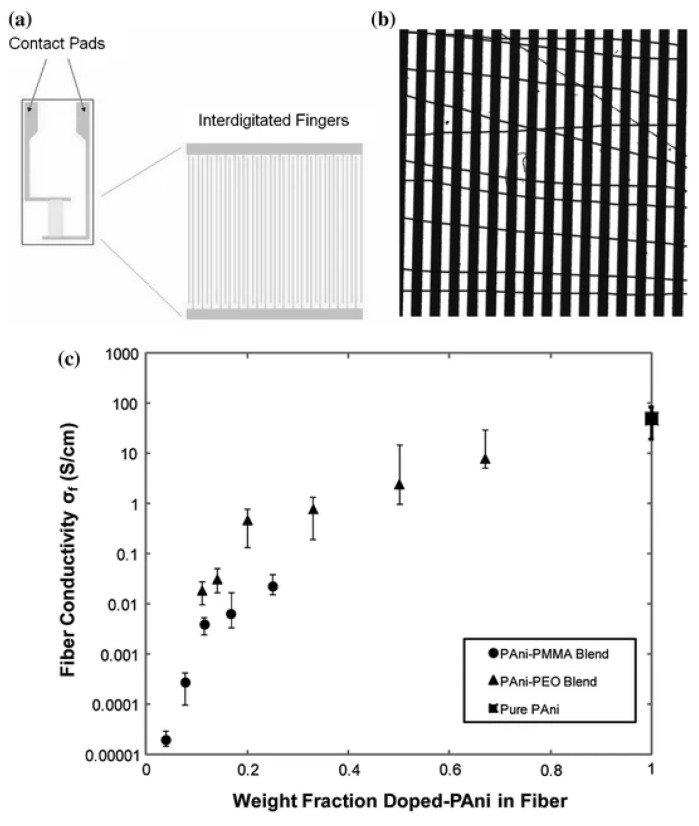
Contact resistance calculation by utilizing overall fiber resistance on interdigitated electrodes (**a**,**b**). Fiber conductivities dependence on weight fraction of doped polyaniline in fibers (**c**). Reproduced from [159] with permission of the American Chemical Society.

Utilizing interdigitated electrodes to measure electrospun nanofibers electrical conductivity is an alternative approach [158]. According to Zhang and Rutledge, electrical contact can be established by applying hot pressure of nanofibers on electrodes [159]. Contact resistance is calculated by determining overall fiber resistance on interdigitated electrodes having varying finger spacing and with the help of extrapolating resistance value at zero spacing, as shown in Figure 23a,b. It is observed that conductivities of fibers increased exponentially by weight fraction of doped polyaniline in fibers; the value for completely doped electrospun fiber was 50 ± 30 S/cm and increased to 130 ± 40 S/cm on further solid state drawing (Figure 23c) [159].

Electrospinning of electrically conductive polymers primarily focuses on polyaniline and its blends. Extremely conductive electrospun polyaniline fiber doped in sulfuric acid is produced by a blend of polyaniline and various conventional polymers such as polystyrene, polyacrylonitrile, polyethylene oxide, etc. [160].

### 5.5. Optical Properties

**Figure 24:**
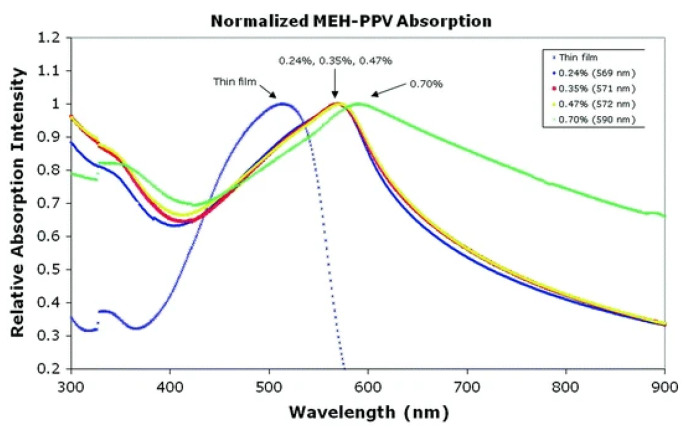
MEH-PPV absorptions of 0.24, 0.35, 0.45, and 0.7% concentrations in chloroform. All curves were normalized to their maximum value. Reproduced from [161] under a Creative Commons Attribution License.

Nagata et al. conducted a lot of optical characterization techniques utilizing a luminescence spectrofluorometer and ultraviolet visible spectrophotometers to study optical characteristics of poly (2-methoxy- 5-(2-ethylhexyloxy)-1,4-phenylenevinylene) electrospun nanofibers [161]. A remarkable red shift was detected at all concentrations of MEH-PPV electrospun nanofibers in comparison to thin film, as depicted in Figure 24. Photoluminescence readings validated the red shift by raising polymer concentration. Babel et al. observed the electrospun nanofiber’s optical properties of conjugated polymer blends utilizing ultraviolet visible spectrophotometers, near-infrared spectroscopy, and photoluminescence spectroscopy [162]. Their finding shows that conjugated polymers’ binary blend has an adjustable composition based on optical characteristics that can be exploited in field-effect transistors. Balderas et al. observed analogous absorption of red shift electrospun fibers generated from a blend of poly(9-vinylcarbazole) and MEH-PPV [163].

### 5.6. Effect of Geometrical Properties and Processing Parameters

To improve the mechanical behavior of a single electrospun nanofiber, it is viable to corelate mechanical characteristics to their geometrical and material properties and processing parameters. Better understanding and proper controlling of mechanical properties of electrospun nanofibers optimizes their characterization so they justify functional and structural application requirements.

#### 5.6.1. Effect of Diameter

**Figure 25:**
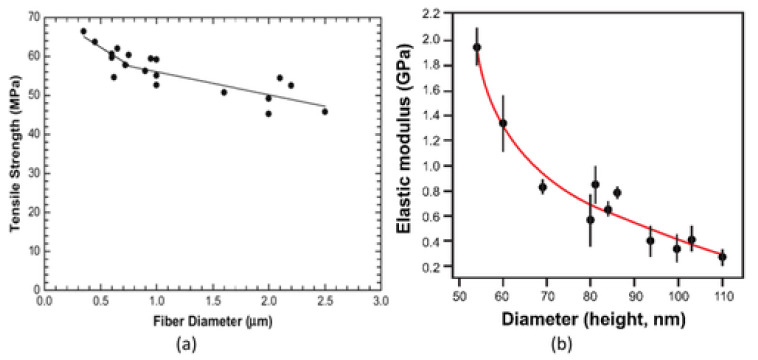
(**a**) Tensile strength and tensile modulus versus diameter. (**b**) Variation in measured elastic modulus as a function of diameter for poly(2-acrylamido-2-methyl-1-propanesulfonic acid) nanofiber. Reproduced from: (**a**) [164] with permission of Elsevier and (**b**) [165] with permission of AIP Publishing.

Experimental investigation of mechanical properties of electrospun polymer nanofibers has shown that they are fundamentally distinct from those of their bigger diameter counterparts. When an electrospun polymeric nanofiber diameter is less than a threshold value, its ultimate tensile strength and axial modulus increase abruptly. Figure 25 shows this observation for a typical case [164,165].

Many researchers have investigated the effect of diameter on the mechanical behavior of electrospun nanofibers [166,167,168]. Tan and Lim noticed variations in mechanical characteristics with fiber diameter of poly (l-lactic acid) nanofiber generated by phase separation. For fiber diameters under 350 nm, the elastic modulus was calculated 1.0 ± 0.2 GPa; as fiber diameters increased 350 nm, the elastic modulus decreases. Later on, this effect was observed during electrospinning of polycaprolactone nanofibers. It was observed that by increasing fiber diameter, ductility is improved but yield stress and tensile strength were decreased. By decreasing fiber diameter from 1.7 to 1.03 μm, the ultimate tensile observed almost doubled. Ji et al. investigated the electrospinning process of poly(2-acrylamido-2- methyl-1-propanesulfonic acid) and found that by decreasing the diameter from 110 to 50 nm, the Young’s modulus increases exponentially from 0.3 to 2.1 GPa [131]. Various factors influence the size of polymer nanofibers, i.e., surface tension [169,170,171], formation of semicrystalline and crystalline structures [117,172], chain alignment, etc. However, the influence of surface energy only is not sufficient to comprehend such behavior in the strength and tensile modulus of polymer nanofibers. Additionally, it was investigated that by increasing the diameter, slight improvement in orientation and crystallinity of nanofibers was achieved [168]. This slight increase cannot justify dramatic increases in Young’s modulus of polymer nanofibers [173,174].

#### 5.6.2. Effect of Collector Type and Take-up Velocity

**Figure 26:**
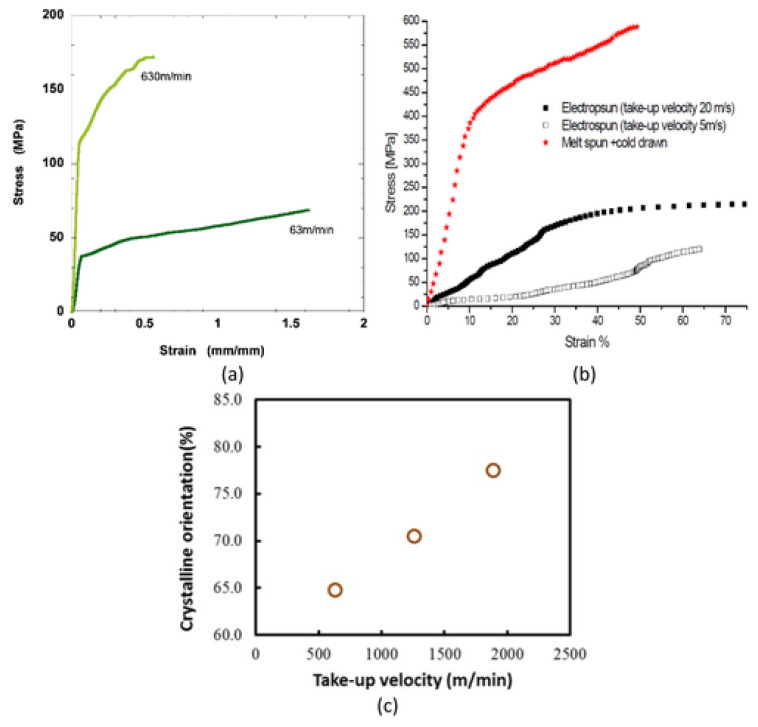
(**a**) Tensile stress–strain curves for PLLA nanofibers at different take-up velocities of 63 and 630 m/min. (**b**) Stress–strain curves of single polyamide-66 (PA-66) nanofibers collected at take-up velocities of 5 and 20 m/s and commercial PA-66 microfiber. (**c**) Crystallinity orientation for take-up velocities of 630, 1260, and 1890 m/min. Reproduced with permission: (**a**) [180] Elsevier, (**b**) [115] AIP Publishing, and (**c**) [181] John Wiley and Sons.

Tensile properties of polymer nanofibers may be significantly influenced by type of the collector. Generally, the web of oriented fibers is accumulated on a rotating disc collector, whereas randomly aligned fibers are gathered on a static collector [175]. Nanofibers with alignment have been shown to have greater tensile strength and modulus than fibers with random orientation, because molecular chains are oriented in the loading direction along the fiber axis. Samples have consequently shown improved tensile properties [37,176]. Tensile modulus and strength were reported to rise with take-up velocity, however, elongation at break decrease as seen in Figure 26a,b, which is mostly attributed to improvement in crystallinity and molecular orientation (Figure 26c) [177,178,179].

**Figure 27:**
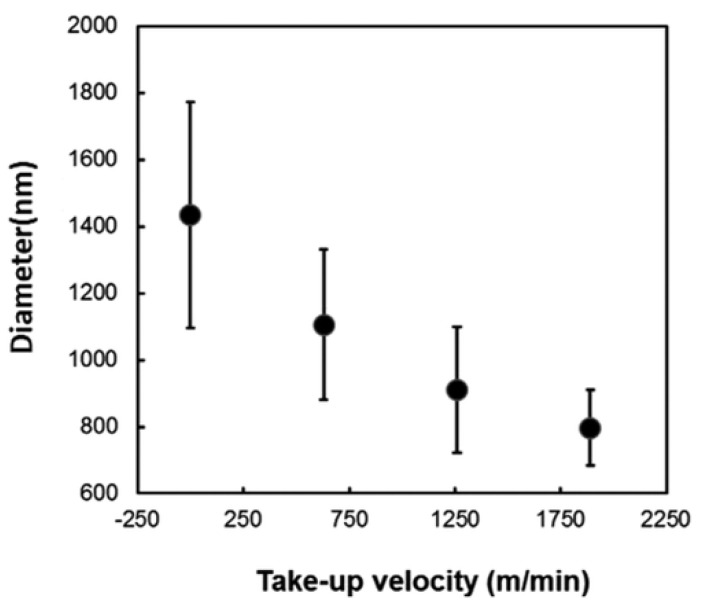
Diameter of porous poly(L-lactic acid) nanofibers as a function of take-up velocity. Reproduced from [181] with the permission of Elsevier.

In addition, increasing take-up velocity results in a reduction in fiber diameter, demonstrating that enhancement pulling force produces alignment and stretching in fibers (Figure 27) [178].

**Figure 28:**
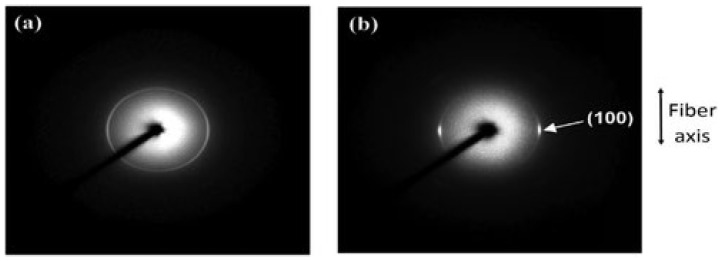
Two-dimensional WAXD patterns for (100) reflection of aligned polyoxymethylene nanofibers at different take-up velocities: (**a**) 630 m/min and (**b**) 1890 m/min. Reproduced from [182] with permission of the American Chemical Society.

Inai et al. studied the influence of take-up velocity on mechanical characteristics of electrospun nanofibers [180]. Poly (L-lactic acid) nanofibers were process by electrospinning at various take velocities, i.e., 63 and 630 m/min, corresponding to disc rotation speed of 100 and 1000 rpm, respectively. Tensile strengths were 89 and 183 MPa for 63 and 630 m/min take-up velocity, respectively. X-ray diffraction analysis shows a highly aligned molecular structure produced by increased take-up velocity. Zussman et al. demonstrates that Young’s modulus of the nylon-6,6 fibers improved from 453 to 950 MPa by increasing take-up velocity from 5 to 20 m/s, employing a rotary collector, which can be explained by the better orientation consistency of electrospun nanofibers at enhanced take-up velocity [115]. It is demonstrated that a greater collecting speed causes a higher degree of alignment in nanofibers (Figure 28) [182].

## 6. Modeling of Electrospun Nanofibers

The superior properties of electrospun nanofibers have contributed to the large interest in processing them and their utilization in industry. The relative simplicity of the electrospinning process made this interest increase. The great effect of the high ratio of surface to volume due to the nano-scale presence gives rise to improved strength, better surface reactivity, larger surface energy, and higher electrical and thermal conductivity. Therefore, seeking to mathematically model the spinning process and resulting properties became of great interest. In what follows, we briefly review some of the most important models in the literature.

### 6.1. One-Dimensional Steady State Model

One-dimensional steady state mathematical model for an electrospinning polymer jet [183,184,185]:

(8) $$Q=\rho \pi ur^{2}$$

(9) $$2\pi ur\sigma +kE\pi r^{2}=I$$

(10) $$u\frac{\partial u}{\partial z}=-\frac{1}{\rho }\frac{\partial P}{\partial z}+\frac{2\sigma E}{\rho r}+\frac{\partial \tau }{\partial z}$$

where E is applied voltage, Q is mass flow rate, P is internal pressure of fluid, ρ is density, *τ* viscous force, r radius of jet at axial coordinate, and z, σ is surface density of charge.

### 6.2. Spivak Dzenis Model

The mathematical model for a steady state polymer jet in the electrospinning process was developed by Spivak and colleagues [186,187,188].

The mass balance equation results in:

(11) ∇·u=0

Linear momentum balance:

(12) $$\rho \left(u\cdot \nabla \right)u=\nabla T^{m}+\nabla T^{e}$$

Electrical charge balance:

(13) ∇·J=0

The right-hand side momentum balance equation is the sum of viscus and electrical forces.

### 6.3. Wan–Guo–Pan Model

The Wan–Guo–Pan mathematical model considers the combined impacts of heat, electricity, modified Navier–Stroke equation, and hydrodynamics considered by this model [189,190]. The governing equations are:

(14) $$\frac{\partial q_{e}}{\partial t}+\nabla \cdot \;J=0$$

(15) $$\rho \frac{Du}{Dt}=\nabla \cdot t+\left(\nabla E\right)\cdot P+\rho f+q_{e}E$$

(16) $$\rho c_{p}\frac{DT}{Dt}=Q_{h}+\nabla \cdot q+J\cdot E+E\frac{DP}{Dt}$$

There are three different current constituents: (a) Ohmic bulk conduction current $I_{c}=\pi r^{2}kE$; (b) current caused by temperature gradient $I_{t}=\pi r^{2}\sigma_{t}\partial T/\partial z$; and (c) surface convection current $I_{s}=2\pi r\sigma u$. To incorporate thermal effect, the above equation can be modified as:

(17) $$\rho \frac{Du}{Dt}=\nabla \cdot t+\left(\nabla E\right)\cdot P+\rho f+q_{e}E+\xi \nabla T$$

### 6.4. Allometric Model

According to Ohm’s law, current flows to the low voltage gradient in proportion to the resistance of circuit. It can be shown that:

(18) $$I=\frac{E}{R}=gE$$

where “*I*” represents the current, *R* the resistance, *E* the voltage, and g the conductance. Ohm’s law is applied to conductors made of metal that have an abundance of electrons. Furthermore, in electrospinning, the current there is not generated by electrons, so the equation should be modified to incorporate characteristics of the polymer. A proposed allometric scaling law for the relationship between conductance and jet radius is as follows [191]:

(19) $$g∼r^{\alpha }$$

where α is a scaling factor.

In the case when α = 2, specimens behave like a metal conductor, and the modified ohm is used for bulk conduction.

(20) $$I_{c}=\pi r^{2}kE$$

where K represents the dimensionless conductivity of the fluid.

In the case when α = 1, there are no free ions or electrons in the bulk, therefore, the current is produced by surface charge, divided along the surface which is in motion. Therefore, surface convection current:

(21) $$I_{S}=2\pi r\sigma u$$

Thus, the value of α will lie between 1 and 2 as conduction of synthesized charged jet falls between Ohmic bulk conduction and surface convection. We can alternatively consider the conductance and polymer concentration scale with each other in the following ways [36,190]:

(22) $$g∼c^{\beta }$$

where β is the scaling exponent, c is the polymer concentration, and β—scaling exponent. Therefore, conductance of an electrospinning jet can be shown as:

(23) $$g=\lambda c^{\beta }r^{\alpha },$$

where λ is constant. In jet, current balance can be shown as:

(24) $$\lambda c^{\beta }r^{\alpha }E+2\pi r\sigma u=I$$

## 7. Applications

### 7.1. Biomedical Applications

One of the most significant applications of polymer nanofibers is in the biomedical sector, particularly in the domains of medication delivery and tissue engineering. Given that biological molecules and nanoscale fibers have similar sizes, the latter are poised to perform well in simulating biological environments and natural extracellular matrices. Nanofibrous meshes exhibit enhanced biological activities, such as increased cell adhesion, differentiation, and proliferation, due to high porosity, large surface area to volume ratio, and interconnectivity of porous matrices comparable to macromolecular ones. Additionally, it is also conceivable for biological molecules to load for nutrients and wastes to exchange through pores [22,192]. The two main research directions in this field are explored below.

#### 7.1.1. Tissue Engineering

**Figure 29:**
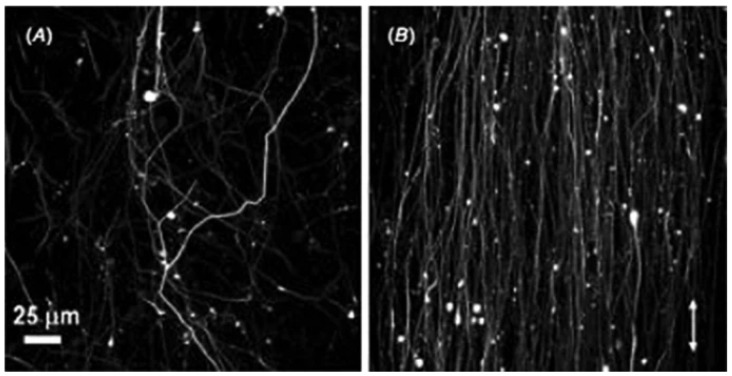
High-magnification confocal microscopy images of neurite morphology on (**A**) random and (**B**) aligned surface modified PLLA nanofibers with BFGF. Neurites oriented in the direction of aligned nanofibers while more neurite branching on random nanofibers was seen. Reproduced from [196] with permission of American Chemical Society.

Electrospun polymer nanofibers utilized as scaffolds in tissue engineering for different tissues such as nerve, bone, blood vessels, cartilage, and skin, etc. have attracted much attention (Figure 29). Restoring harmed tissues is a primary objective of tissue engineering. Due to comparable fibrous structure of nanofibrous made of biodegradable polymers with natural extracellular matrices, these structures operate so as to support cell, proliferation, adhesion, and differentiation. As such, they possess great potential as scaffolds for tissue regeneration [179,193]. Collagen, keratin, elastin fibers, etc. obtained from extracellular matrix are the most often employed materials in this endeavor because they are inherently fibrous in nature and easily transformed into fibrous scaffolds. Polysaccharides, proteins, and various biomedical materials were investigated for scaffold production because of their exceptional features such as water solubility, biodegradability, biocompatibility, water absorption, and hydrophobicity, among others. Natural polymers such as cellulose, amylose, heparin, dextran, glycosaminoglycan, and chitin are widely used compounds for scaffolds. Synthetic polymer materials such as poly caprolactone, poly lactic acid, polyvinyl alcohol, and polylactic co-glycolic acid with ceramic bioactive materials are used for scaffold fabrication [194]. Electrospun polymer nanofibers have demonstrated potential in the reconstruction of specific tissues, but still further improvements are needed in biological compatibility and chemical and mechanical properties, which represent promising areas of interest to further progress [195].

#### 7.1.2. Wound Healing and Dressing

In wound rehabilitation pursuit, the large porosity of electrospun fibers may provide additional structural space for accommodation of transplanted cells, promote cell migration and proliferation, and increase oxygen exchange and waste outflow. The tiny pore size of nanofibrous scaffolds can prevent dehydration and wound infection throughout the healing process. Additionally, tunable mechanical properties of electrospun nanofibers can maintain mechanical consistency between tissue engineering grafts and parent tissue and prevent the wound from wrinkling or shrinkage during implantation.

For skin tissue engineering, various natural and synthetic polymers are electrospun to nanofibrous scaffolds. Natural polymers such as collagen, fibrinogen, chitosan, silk, etc. have been processed for wound healing. Collagen is a principal element of the human skin extracellular matrix; it develops a three-dimensional fibrillar network structure with diameters in the range of 50–500 nm to normalize attachment, differentiation, and proliferation in skin texture. Electrospun cellulose gelatin/acetate nanofibers have resemblance to the extracellular matrix composition of skin. It was studied that 75/25, gelatin/cellulose acetate nanofibers revealed distinctive adhesive features and improved fibroblast proliferation to human skin. However, utilizing natural polymers for tissue engineering revealed poor mechanical properties and less resistivity to enzymatic degradation, which are among major challenges facing this promising technology. A variety of synthetic biodegradable polymers such as polyglycolic acid, polylactic acid, polycaprolactone, and copolymers are generally used for skin tissue engineering because of their advantageous mechanical and biodegradable characteristics. For example, electrospinning polylactic-co-glycolic acid with glycolide/lactide having a molar ratio of (75:25, 85:15), respectively, can accomplish the required biodegradable scaffolds for the replacement of damaged skin. The usage of synthetic polymers is, however, constrained by their hydrophobic surface and limitations of cell-detection signals [197].

*Portable Electrospinning for Wound Dressing*

An important promising development is in portable electrospinning technology. Portable electrospinning works by directly producing healing nanofibers on the wound to customize the dressing, relieve the pain, and increase the correspondence to wound bed. These features give it the advantage over using pre-prepared nanofibers in different types of wounds such as cut skin, irregular skin wounds, burned skin, liver cuts, dural repairs, etc. Furthermore, it also leads the way for personal wound dressing. Practically, in situ wound dressing is possible by a portable electrospinning setup that is user friendly and safe to operate. A portable electrospinning machine was utilized for in situ spinning of poly (lactic-co-glycolic acid) fibers in the wound dressing process, where they were deposited precisely on the injured areas [19].

For in situ electrospinning, the versatility of portable devices depends upon biological collector and modified components of devices such as spinneret and voltage supply systems. The portability of devices depends upon the requirements and nature of modifications. Portable devices have different sources of power supply, which are summarized in Table 5.

#### 7.1.3. Drug Delivery Systems

Various drug delivery systems such as polymer micelles, liposomes, and nanofibers are studied to diminish the toxicity of dosage and increase therapeutic efficacy [198,199,200,201]. There is a potential for electrospun nanofibers to provide significant benefit due to flexibility in selection of materials and medications, encapsulation efficiency, and delivery of therapeutic agents, among others, which makes them appealing candidates in drug delivery, particularly for topical chemotherapy after surgery and in wound casing materials [37]. Electrospun nanofibers are utilized in precise and localized drug delivery systems thanks to their main advantages of large surface to volume ratios and well-interlinked, open porous structure. Numerous attempts have been undertaken to integrate bioactive compounds after electrospinning them, either chemically or physically, into the scaffolds. As per one reference study, the easiest approach to entrap biomolecules to electrospun nanofibers is the dip-coating process. Alternatively, the polymer solution is mixed with a bioactive molecule to generate a composite by blend electrospinning [195]. Techniques such as blending, co-axial electrospinning, and surface modification are utilized to load drugs into nanofibers. However, when developing a scaffold for drug eluting, some critical factors such as biodegradability, biocompatibility, adequate mechanical characterization, and proper amount of drug are to be considered carefully. Mechanical properties, drug release kinetics, and biodegradability are adjusted by proper selection of polymers and parameters effecting the electrospinning process, while biocompatibility is tuned through surface modifications techniques.

### 7.2. Sensors and Biosensors

Nano-based sensors and biosensors are utilized according to combined chemical and physical principles based on the two main components of the receptor and the transducer. The receptor reacts chemically to the change and creates a form of energy that the transducer measures and signals [202]. Significant advancements have been achieved in the manufacturing of extremely biological and chemical sensitive sensors in response to increasing demands for high-precision reliable detections in different and evolving applications in medicine and sophisticated manufacturing for targeted industries. Electrospun polymer nanofibers provide a fertile source of utilization in sensing applications. An optical sensor that was developed by the electrospinning of fluorescent polymer nanofibers showed three-time improved sensitivity magnitude as compared to film sensors for detection of mercury ions and nitro and ferric compounds. Conductive electrospun polymer nanofibers such as polyaniline nanowires are strong candidates for sensing applications due to their outstanding electrical characteristics [203,204,205]. A nanofibrous system having a diameter below 20 nm exhibits extraordinarily high porosity, large surface area, and superior mechanical characterization, making them ideal candidates for ultrathin filters and ultrasensitive censoring. Recently, gas sensor technology boosted up due to the urgent need to combat increasing environmental pollution through the detection of gas structures to identify the exact gas based on different sensing techniques. Various electrospun polymer nanofibers are being used as detecting interface agents to sense variety of gases. In a hydrogen sensing experiment, the electrode is modified with an electrospun polyvinylpyrrolidone/lithium tantalum oxide composite nanofiber result in faster response and higher sensitivity of hydrogen gas as compared to a film-based sensor [206,207].

### 7.3. Air Filtration

Responding to air pollution challenges, which stem basically from especially fine particle in the environment, has become a pressing priority, whether in industrial sites or in urban areas. With the increasing pace of industrialization and energy consumption and mining, the urgency is even greater. Volatile organic compounds and microbes in air can seriously harm human life [208]. Various studies have suggested that electrospun nanofibers have the ability to capture such volatile organic compounds in air. Electrospun polymer nanofiber membranes have shown faster adsorption and desorption of volatile organic compounds compared to conventional activated carbon. The performance of filter membranes is significantly influenced by the structural properties of electrospun fibrous membranes. Fiber diameter and distribution, pore size distribution, surface area, basis, and density constitute the determining factors for filtering process effectiveness. According to B. Maze et al., polymer nanofibers with lesser diameter will have a more accessible surface area, which will reduce pressure drop. Therefore, selecting an optimal electrospun nanofiber diameter is essential to maximizing filtration performance. It has been investigated that stacking multiple electrospun nanofiber membranes having lower basis weights is much more efficient than using a single layer of electrospun nanofibrous membrane having a high basis weight. According to a study by Kim et al., appropriate electrospun nanofibrous film thickness influences the efficiency of the air filtration medium, but extremely thick films would reduce air filtration performance due to enhanced pressure drop through filtration media. Along with these structural characteristics, other environmental and testing factors such as face velocity, temperature, particle size, and humidity have a significant influence on filtration performance [208].

### 7.4. Defence Applications

Polymer nanofibers are considered as exceptional membrane materials for the defense industry in smart textiles for the detection of biological and chemical warfare agents with high sensitivity. Their high sensitivity to biological and chemical pollutants at concentrations of parts per billion make them attractive candidates for sensing interfaces for warfare agents [209]. In one case, a PVC nanofiber was used as a mount for detoxifying agents capable of high efficiency to trap chemical warfare agents. High porosity and hydrophilicity of the NF demonstrated their suitability as a filtering medium in this application [210]. Other investigations were conducted to incorporate nanoparticles into an electrospun nanofiber to enhance their properties for effective utilization in smart textiles. In electrospinning polymers such as polyamide, polypropylene, polyvinyl alcohol, etc., their composites are utilized. Mechanical, thermal, and physical properties of nylon 6 or polyamide can be improved by integrating nanoparticles of magnesium oxide, silicon dioxide, titanium dioxide, zinc oxide, and zirconium dioxide. Moreover, nanometal oxides are employed as a matrix to improve properties such as ultraviolet protection, anti-flammability, and antibacterial activity [211].

### 7.5. Energy Devices

Renewable energy resources are becoming more dominant to ensure economic growth due to the diminution of fossil fuels and huge increase in demand for energy. It was reported that nanofibers perform better than typical materials in devices for energy storage, harvesting, and conversion, offering good alternative materials for use in energy devices such as lithium-ion batteries, nanogenerators, and solar cells [6]. Nanofibers used in solar cells have shown high photoelectric conversion efficiency due to separation, efficient charge transmission, and high light absorption mainly due to a large specific surface area and high porosity. The large ratio of surface area to volume in nanofibers enhances formation of the nonwoven structure, which improves conductivity and gives rise to possible utilization of NF in batteries and fuel cells as a separation medium. Nanofiber-based electrodes in solar cells have shown high cycling stability and specific capacity. Enhancement in hydrogen production due to photocatalytic action of electrospun polymer nanofibers as photocatalysts in water splitting. Furthermore, it is important to note that lithium-ion batteries, with their unique features such as a long-life cycle and high energy density, have paved the way to more improvements in energy storage technologies. This will definitely be enhanced by the development of the utilization of nanofibers in lithium-ion batteries because of their superior electrochemical performance, mechanical strength, and enormous specific surface area [212].

To conclude this section on applications of polymer nanofibers, the mechanisms by which different polymer nanofibers act to achieve the needed outcome in different applications are summarized in Table 6.

### 7.6. Commercialization

The versatile applications for electrospinning of polymer nanofibers stem from the quite diverse sets of technologies employed, polymers and solvents used, conditions of processing … etc. In all applications, there are the driving forces of market volume, demand, and how critical the application is. The medical field is a strong market, but there are also other critical applications such as water treatment and air filtration, in addition to textile and other industries. Table 7 summarizes most notable commercial utilization of polymer nanofibers in various fields.

## 8. Conclusions

Due to their unique characteristics, ease of production, and diversity of combinations, in addition to superior properties, electrospun polymer nanofibers (EPNF) are poised to play a larger role in the development of many technologies that span different areas of human demand in different sectors. In this paper, we presented an encompassing review of polymer nanofibers’ attractive features, production techniques, and, especially, electrospinning, including a detailed description of the process. The polymers that are used in electrospinning were covered. Special coverage of the most important electrospinning process parameters was included so that the processes’ different variations can be understood through manipulation of such parameters. EPNF mechanical and other properties of EPNF were reviewed, with special attention to mechanical tests and techniques. Due to the importance of models in predicting the features and behavior during processing and in service, a brief review of important mathematical models was also presented. The increasing and widening applications of EPNF were included in the final section, covering various fields from biomedical applications, sensing, and biosensing technologies to defense and energy applications.

The field is ripe for more advances in research and industrial endeavors, due to the interesting findings in both diversification of processing technologies and techniques and also in the lateral and vertical expansion of applications. More efforts should be made in the experimental, computational, and theoretical directions. With the wide choices for the direction of experimental research, the need is great for the development of theoretical and computational models to streamline choices of experimentation, whether in the choice of pristine polymers or processing techniques and parameters. The ability to characterize features and behavior, even with some degree of idealization and simplifications, will offer better informed choices for development directions in the experimentation to improve electrospinning processes’ efficiency and precision and directions in which to expand and improve utilization throughout the wide spectrum of applications.

## Figures and Tables

**Table 1:** Different techniques for polymer nanofibers production, from different sources.

Technique	Principle	Pot ^1^	Rep ^2^	FDC ^3^	Advantages (+)/Limitations (−)	Refs.
Phase separation	Polymer rich and poor phases are generated, elimination of poor phase leads to generation of nanofibrous structure	Lab	Yes	Yes	+ Consistent for batch-to-batch production, can adjust props by varying concentration + Easy − Few polymers can be processed	[13,14]
Drawing	Pulling, followed by solidification, of dissolved spun material to solid material	Lab	Yes	No	+ Less equipment required for processing + Easy processing − Discontinuous, fibers generated sequentially	[15]
Self-assembly	Used to produce peptide nanofibers	Lab	Yes	No	− Few polymers can be processed −complex − Difficult	[16]
Template synthesis	Nano-porous membrane is utilized as template with different materials (metals, carbon, conductive polymers etc.)	Lab	Yes	Yes	+ Fibers of different diameters can be produced using various templates + Moderately easy − Applicable for few polymers	[17]
Electrospinning	Electric field to process micro/nanofibers from polymer in liquid solution of melts	Lab and comm.	Yes	Yes	+ Simple, uses various polymers, produces long continuous nanofibers, feasible to generate aligned nanofibers + Moderately easy	[19,20]

^1^ Potential of the process. ^2^ Repeatability. ^3^ Fiber dimensional control.

**Table 2:** Polymer nanofibers electrospinning techniques.

Technique	Principle	Advantages/Limitations
Melt Electrospinning	Apparatus is similar to conventional electrospinning setup along with provision of melting of polymer.	+ Environmentally friendly without need for ventilation system. + High throughput rate and easily processed polymeric fiber blends. + Suitable to electrospin non-soluble polymers (such as PE and PP). − Require high temperature melting system, electric discharge issues related to melt, and low conductivity of melt. [42]
Needleless/free surface electrospinning	Simultaneous generation of various jets from open liquid surface without capillary effect.	Classified into rotating and stationary needleless electrospinning. Morphology and production rates depend on shape of spinneret (conical wire coil, bowel edge, rotary cone, metal plate, splashing, and moving bead chain) + Production rates bet. 2.5–100 g/h are possible, up to 250 times greater than conventional spinning. − Difficult to control the spatial movement of multiple jets and inadequate stability of the free liquid. [43]
Multi-jet Electrospinning	Polymer is split up into distinct jets during trajectory.	Needles are arranged linearly or in 2D with triangular, circular, square, elliptical, and hexagonal arrays. + Upscale electrospinning process by utilizing multiple needles. − Interference of electric fields − Clog up of needles with polymer solutions. − Jet deviation causes instability issues, requiring auxiliary plate and extra electrode configurations. [44]
Electro-blowing (Gas Jet/Gas Assisted Electrospinning)	Air blowing shear force and electric field simultaneously applied to generation of nanofibers.	A spinneret with air nozzle generates nanofiber web to electrospin thick solutions where surface tension is very high. + Improved productivity + Hot gases can be utilized to reduce solution viscosity and increase the fiber elongation. [45]
Centrifugal Electrospinning	Combination of electrical and centrifugal forces in electrospinning process.	+ Rotation range: 300–600 rpm, << centrifugal spinning range. + Improved polymer chain orientation and production. + Due to centrifugal effect, projection force on jet is higher, resulting in less flight time and improving the fiber stretching. + High production rate, easy maintenance. − Non continuous fibers, requires expensive equipment. [46]
Near-Field Electrospinning	In this technique, distance between substrate and nozzle is reduced few millimeters.	+ Utilized for deposition of nanofibers at predictable location, which is not feasible by common electrospinning process. + Lower voltage (~ 200 V) is required to generate electric field to initiate spinning. + Improvement in morphology of nanofibers. [47]
Coaxial Electrospinning	Utilized two concentrically align capillaries to generate core shell structure.	+ The setup can be configured in both vertical and horizontal direction. + It can generate hollow electrospun nanofibers. + Almost all polymer solutions and their composites’ matrices can be processed into core shell and hollow nanofibers. − Dimensional control is difficult, solvent evaporation issues. [48]
Emulsion electrospinning	Similar to solution electrospinning, but solution is substituted by emulsion with oil in water or water in oil.	+ Used to generate core-shell fiber, it is a relatively simpler setup than co axial electrospinning. + Utilized for encapsulation of range of bioactive materials with various solubilities into polymeric nanofibers. + User friendly and economical technique. [49]

**Table 3:** Polymers used in electrospinning, and their characterization techniques and applications.

Polymers	Solvents	Applications	Refs.
Nylon6,6, PA-6,6	Formic acid	Smart clothing	[60]
Polylactic acid	Dimethyl formamide Methylene chloride and dimethyl Formamide Dichloromethane	Membrane for medical use, sensor, filter, drug delivery system	[7,11]
Polybenzimidazole	Dimethyl accetamide	Protective clothing, nanofiber reinforced composites	[61]
Polycarboate	Dimethyl ormamide: tetrahydrofuran (1:1), Dichlormethane, chloroform, tetrahydrofuran Dimethylformamide: tetrahydrofuran (1:1)	Protective clothing, sensor, filter	[62]
Polyacrylonitrile	Dimethyl formamide	Carbon nanofiber	[63]
Polyurethanes	Dimethyl formamide Dimethylformamide	Protective clothing Electret filter	[64,65]
Polyvinil alcohol	Distilled water		[66]
Polyethylene-co-vinyl acetate		Drug delivery system	[67]
Polyethylene oxide	Distilled water Distilled water: ethanol (3:2) Isopropyle alcohol+water	Microelectronic wiring, interconnects Electret filter	[68]
Collagen–Polyethylene oxide	Hydrochloric acid Hydrochloric acid (pH = 2.0)	Wound healing, tissue engineering, hemostatic agents	[69]
Polyaniline/polyethylene oxide blend	Chloroform, camphorsulfonic acid	Conductive fiber	[70]
Polyaniline/polystyrene	Chloroform, camphorsulfonic acid	Conductive fiber	[71]
Silk-like polymer with fibronectin functionality	Formic acid	Implantable device	[72]
Polyvinylcarbazole	Polyvinylcarbazole	Sensor, filter	[73]
Polystyrene	Tetrahydrofuran, dimethylformamide, CS2 (carbon disulfide), toluene, Methylethylketone, Dimethylformamide Tetrahydrofuran	Enzymatic biotransformation, Flat ribbons, catalyst, filter	[11]
Polyamide	Dimethylacetamide	Glass fiber filter media	[74]
Silk/polyethylene oxide blend	Silk/PEO blend	Biomaterial scaffolds	[75]
Poly vinyl phenol	Tetrahydrofuran	Antimicrobial agent	[76]
Antimicrobial agent	Acetone, acetic acid, dimethylacetamide	Membrane	[77]
Mix of (polyacrylic acid -polypyrene methanol) and polyurethane	Dimethylformamide	Optical sensor	[78]
poly(lactic-co-glycolic acid)	Tetrahydrofuran: dimethylformamide (1:1)	Scaffold for tissue engineering	[79]
Collagen	Hexafluoro-2-propanol	Scaffold for tissue engineering	[80]
Poly (vinylidene fluoride) PVDF	Dimethylformamide: dimethylacetamide (1/1)	Flat ribbons	[81]
Nylon-4,6, PA-4,6	Formic acid	Transparent composite	[82]
Transparent composite	Isopropanol/water: 70/30 (%*v*/*v*)	Biomedical	[83]
Polyacrylnitrile/TiO_2_		Photovoltaic and conductive polymers	[84]
Polycaprolactone/metal		ZnO: cosmetic use	[85]

**Table 4:** Summary of parameters effecting morphology of electrospun nanofiber.

Different Parameters	Effect on Morphology
↑ Polymer concentration	↑ Fiber diameter if > 15 wt.% (with in optimal range) ↓ Bead formation
↑ Molecular weight	↓ Droplet and bead formation Irregularity in shape with larger pores
↑ Volatility of solvent	Pores generated on surface of fiber (macrotexture)
↑ Solution conductivity	↓ Fiber diameter (broad distribution of diameter) and uniform bead free fibers
↑ Distance between collector and capillary	↓ Fiber diameter, for generation of uniform fiber optimal distance, i.e., 30 cm is required, too short or too long cause bead formation
↑ Voltage applied	↓ Fiber diameter initially, then ↑ (not monotonic)
↑ Feed rate	↑ Fiber diameter and, if too high, bead formation occurs
↑ Temperature	↓ Fiber diameter and ↓ viscosity
↑ Air velocity	↑ Fiber diameter
↑ Humidity	Produce circular pores on fiber

**Table 5:** Sources of power for portable electrospinning devices.

Type of Portable Devices	Advantages/Limitations
Hand-held spinnerets	+ Flexible in operation, precise deposition of fibers, accuracy in flow rate, in situ spinning. − Electricity dependent, expensive.
Generator powered	+ Power supply, in situ spinning, flexible in operation, affordable. − Unstable voltage, not precise deposition, limited flow rate.
Battery powered	+ Precise deposition, flexible in operation, precise deposition, affordable. − Battery capacity issues, high voltage limitation.

**Table 6:** Nanofibers’ mechanisms of action in different applications.

Application	Action/Mechanism
Tissue Engineering	NFs act as scaffolding for tissue regeneration and growth.
Wound Healing and Dressing	NF porosity provides space for cell migration and regeneration and increases oxygen exchange, in addition to reinforcement.
Drug Delivery Systems	Porosity, large aspect ratio, and cross-linking give rise to drug-carrying capacity at minute volumes for directed delivery.
Sensors and Biosensors	The NF chemically reacts to the change and activates an energetic signal to be detected and recorded.
Air Filtration	NF high cross-linking and spatial structure provide ability to capture microparticles and filter the environment.
Defense: protection from warfare toxic gasses	NFs carry catalysts capturing toxic warfare chemical stimulants. Porosity and hydrophilicity of membranes qualify them as filter media.
Energy Devices	NFs are used as separators in fuel cells and large photoelectric conversion efficiency due to large specific area and porosity.

**Table 7:** Commercial nanofiber products utilized in various fields.

Product	Utilization	Manufacturer	Description	Websites ^1^
Aeos	Wound Healing	Zeus Company Inc.	Consist of nonwoven fibrous for sutures	https://www.zeusinc.com/
ResQFoam	Wound Healing	Arsenal Medical	Non-compressible hemorrhage treated by foam contained in core shell fiber	https://arsenalmedical.com/
Rethink	Surgical mask	Stellenbosch Nanofiber Company	Specializing in commercial scale manufacture of advanced biomedical nanofiber materials	https://sncfibers.com/
Spincare	Wound Healing	Nanomedic	Portable electrospinning equipment	https://nanomedic.com/
Nexture	Textile	Lime Nano	Nanofibers membranes	https://limenano.com/
NanoDream	Textile	NanoLayr	Nanofiber clothing	https://www.nanolayr.com/
Wetlaid Fabrics	Textile	Hirose Paper Mfg. Co	Nanofiber Nonwoven Fabrics	https://www.hirose-paper-mfg.co.jp/
BreaSAFE	Air Filtration	Nano4Fibers	Masks are made of nanofibers to protect from microbes, toxic gases, dust, and order.	https://www.nano4fibers.com/
FilterLayr	Air Filtration	Nanolayr	Air filtration unit	https://www.nanolayr.com/
Exceed	Air Filtration	Espin Technologies, Inc.	Nanofiber membranes for air filtrations	https://espintechnologies.com/
ProTura	Water treatment	Parker	Nanofibers cellulose filtration assembly	https://www.parker.com/
Nanofiber Filter	Water Treatment	Astral Pool	Self-cleaning filters	https://www.astralpool.com/

^1^ All websites accessed on 6 December 2022.

## Data Availability

Not applicable.
